# Candidate Markers for Stratification and Classification in Rheumatoid Arthritis

**DOI:** 10.3389/fimmu.2019.01488

**Published:** 2019-07-05

**Authors:** Lucius Bader, Stein-Erik Gullaksen, Nello Blaser, Morten Brun, Gerd Haga Bringeland, André Sulen, Clara Gram Gjesdal, Christian Vedeler, Sonia Gavasso

**Affiliations:** ^1^Bergen Group of Epidemiology and Biomarkers in Rheumatic Disease, Department of Rheumatology, Haukeland University Hospital, Bergen, Norway; ^2^Department of Clinical Science, University of Bergen, Bergen, Norway; ^3^Center of Cancer Biomarkers, University of Bergen, Bergen, Norway; ^4^Department of Internal Medicine, Hematology Section, Haukeland University Hospital, Bergen, Norway; ^5^Department of Mathematics, University of Bergen, Bergen, Norway; ^6^Department of Neurology, Haukeland University Hospital, Bergen, Norway; ^7^Department of Clinical Medicine, University of Bergen, Bergen, Norway

**Keywords:** rheumatoid arthritis, patient stratification, tumor necrosis factor, tumor necrosis factor inhibitors, mass cytometry

## Abstract

Rheumatoid arthritis (RA) is a chronic autoimmune, inflammatory disease, characterized by synovitis in small- and medium-sized joints and, if not treated early and efficiently, joint damage, and destruction. RA is a heterogeneous disease with a plethora of treatment options. The pro-inflammatory cytokine tumor necrosis factor (TNF) plays a central role in the pathogenesis of RA, and TNF inhibitors effectively repress inflammatory activity in RA. Currently, treatment decisions are primarily based on empirics and economic considerations. However, the considerable interpatient variability in response to treatment is a challenge. Markers for a more exact patient classification and stratification are lacking. The objective of this study was to identify markers in immune cell populations that distinguish RA patients from healthy donors with an emphasis on TNF signaling. We employed mass cytometry (CyTOF) with a panel of 13 phenotyping and 10 functional markers to explore signaling in unstimulated and TNF-stimulated peripheral blood mononuclear cells from 20 newly diagnosed, untreated RA patients and 20 healthy donors. The resulting high-dimensional data were analyzed in three independent analysis pipelines, characterized by differences in both data clean-up, identification of cell subsets/clustering and statistical approaches. All three analysis pipelines identified p-p38, IkBa, p-cJun, p-NFkB, and CD86 in cells of both the innate arm (myeloid dendritic cells and classical monocytes) and the adaptive arm (memory CD4^+^ T cells) of the immune system as markers for differentiation between RA patients and healthy donors. Inclusion of the markers p-Akt and CD120b resulted in the correct classification of 18 of 20 RA patients and 17 of 20 healthy donors in regression modeling based on a combined model of basal and TNF-induced signal. Expression patterns in a set of functional markers and specific immune cell subsets were distinct in RA patients compared to healthy individuals. These signatures may support studies of disease pathogenesis, provide candidate markers for response, and non-response to TNF inhibitor treatment, and aid the identification of future therapeutic targets.

## Introduction

Rheumatoid arthritis (RA) is a chronic autoimmune disease characterized by synovial inflammation that, if not treated early and efficiently, causes joint damage. The pro-inflammatory cytokine tumor necrosis factor (TNF) plays a central role in the pathogenesis of RA and is the target of treatment with TNF inhibitors. TNF inhibitors are generally effective and well-tolerated (1, 2); however, up to one-third of patients are primary non-responders, and responses in up to one-third of initial responders abate over time (3, 4).

Currently, only a few markers for diagnostic and stratification purposes are used in daily clinical practice in patients with RA. Anti-citrullinated peptide antibodies are a highly disease-specific biomarker with an impact mostly on diagnosis and classification (5). TNF inhibitor drug levels and anti-drug antibodies are indicative of treatment responses; however, these markers are not standardized for clinical application (3, 6, 7). Several candidate biomarkers for prediction of treatment responses have been suggested based on gene, cytokine, and immune cell profiles, but none have added significant value to patient stratification in a clinical setting (8). Previous studies have indicated the potential of single-cell profiling by flow or mass cytometry in patient stratification in RA and in other autoimmune conditions (9, 10). Distinct signaling patterns have been found in RA patients before and during treatment with TNF inhibitors in exploratory and proof-of-principle studies (11, 12).

We hypothesize that signaling patterns in RA are distinct from those of healthy donors. The unbiased identification of RA-specific signaling patterns in immune cell subsets before treatment may improve diagnosis, therapeutic stratification, and monitoring, and may also facilitate studies of disease pathogenesis and the development of drugs that target dysfunctional pathways with high precision.

In this study, we used mass cytometry to explore signaling responses to TNF in single immune cells of RA patients and healthy donors. In mass cytometry metal-tagged antibodies serve as markers with a read-out in a mass spectrometry time-of-flight chamber (13). Using mass cytometry, up to 50 markers can be simultaneously analyzed with single cell resolution with relatively little signal overlap and very low background noise (14, 15). Here we used a panel of 13 phenotyping and 10 functional markers for an in-depth characterization of peripheral blood mononuclear cells (PBMCs) from patients and controls with and without stimulation with TNF. Based on results from three different analysis pipelines, we suggest a smaller set of phenotyping and functional markers, which strongly correlate with disease status for future use in e.g., flow cytometry.

## Materials and Methods

In-depth information on material, methods and results is provided in the Supplementary Material in the same order and with the same headings/sub-headings as in the main article.

### Healthy Donors and RA Patients

Twenty healthy donors (HD, 4 male, 16 female, ages 39–67) and 20 RA patients (4 male, 16 female, ages 31–76) were included in this study (Table 1). All RA patients were included at the time of diagnosis and fulfilled the ACR/EULAR 2010 criteria for RA. None of the patients had received synthetic or biologic disease-modifying anti-rheumatic drugs, but five had been prescribed low to moderate dosages of prednisolone by their general practitioners prior to the first consultation with a rheumatologist. Despite ongoing prednisolone-treatment at inclusion (range 2.5–15 mg), these patients had high disease activity with a mean disease activity score (DAS28) of 6.1 (range 5.4–7).

**Table 1:** Patient and healthy donor characteristics.

**Healthy donors (HD)**	
Female/male	16/4
Median age (range)	49 (34–67) years
**RA patients (RA)**	
Female/male	16/4
Median age (range)	63.5 (31–76)
**Disease characteristics**	
RF+	14
ACPA+	11
RF+ ACPA+	9
RF- ACPA-	4
Mean DAS28 (range)	5.37 (3–7.6)
Mean DAS28–CRP (range)	4.86 (2.5–7.2)
Mean CRP (range)	25.3 (1–156)
Mean ESR (range)	36.6 (6–104)
**Medication**	
Prednisolone	5 of 20 patients
Prednisolone daily dose	2.5-15 mg (2.5, 2.5, 12.5, 12.5, 15 mg)

*RF, rheumatoid factor; ACPA, anti-citrullinated peptide antibodies; DAS28, disease activity score with 28 joint count; CRP, C-reactive protein; ESR, erythrocyte sedimentation rate*.

All donors and patients gave written informed consent for inclusion into the Norwegian Arthritis Registry (NorArtritt) and the Research Biobank for Rheumatic Diseases in Western Norway (approval REK 2012/1689). Utilization of registry data and biobank material for this study was approved by the Regional Ethics Committee (approval REK 2014/317).

### Peripheral Blood Mononuclear Cells (PBMCs)

PBMCs were chosen due to the possibility of culturing and application of standardized and simultaneous conditions (such as e.g., cytokine stimulation) after cryo-preservation.

PBMCs were harvested by density gradient centrifugation (BD Vacutainer® CPT™ Mononuclear Cell Preparation Tube—Sodium Citrate), processed for cryo-preservation within 4 h and stored in liquid nitrogen in 50% hematopoietic cell medium (X-VIVO™, Lonza), 42.5% freezing medium (ProFreeze™, Lonza), and 7.5% dimethyl sulfoxide (Sigma-Aldrich).

### Antibody Panel

All antibodies used in this study (Table 2) were titrated on PBMCs from one healthy donor. Titrations were performed on unstimulated PBMCs and cells stimulated with TNF and phorbol myristate acetate (PMA) for optimization of pathway activation markers. The antibodies against CD120a, CD120b, and p-cJun were conjugated to metals in our laboratory (conjugation kits and protocols by Fluidigm), all other antibodies were pre-conjugated (Fluidigm).

**Table 2:** Antibody panel with epitopes, antibody clones, conjugated metals, and target cell populations or signaling pathways.

**Epitope**	**Clone**	**Metal**	**Target/Function**	**Abbrev**.
**PHENOTYPING**				
CD20	2H7	^147^Sm	B lymphocytes	Bc
CD3	UCHT1	^170^Er	T lymphocytes	
CD4	RPA-T4	^145^Nd	CD4^+^ T lymphocytes	CD4 Tc
CD8a	RPA-T8	^146^Nd	CD8^+^ T lymphocytes	CD8 Tc
CD45RA	HI100	^169^Tm	Naïve/effector vs. memory	Naïve, mem
CD56	NCAM16.2	^176^Yb	Natural killer cells	NKc
CD16	3G8	^148^Nd	NK T cells	NK Tc
CD14	M5E2	^160^Gd	Classical monocytes	cM
CD61	VI-PL2	^209^Bi	Monocytes	cM
CD11c	Bu15	^159^Tb	Myeloid dendritic cells	mDc
CD123 (IL-3R)	6H6	^151^Eu	Plasmacytoid dendritic cells	pDc
HLA-DR	L243	^174^Yb	MHCII, antigen presentation	
CD45	HI30	^89^Y	Leukocyte Common Antigen	
**FUNCTIONAL**				
Cleaved Caspase 3	D3E9	^142^Nd	Apoptotic signaling	Caspase3
p-p38 [T180/Y182]	D3F9	^156^Gd	MAPK pathway	p-p38
p-Erk1/2 [T202/Y204]^*^	D13.14.4E	^171^Yb	MAPK pathway	p-Erk
p-Akt [S473]	D9E	^152^Sm	PI3K-Akt pathway	p-Akt
p-cJun [S73]^**^	D47G9	^167^Er	SAPK/JNK signaling	p-cJun
p-NFkB p65 [S529]	K10-895.12.50	^166^Er	NFkB canonical pathway	p-NFkB
IkBa	L35A5	^164^Dy	with IkBa degradation	IkBa
CD120a^**^	MABTNFR1-B1	^155^Gd	TNF receptor 1	TNFR1
CD120b^**^	hTNR-M1	^165^Ho	TNF receptor 2	TNFR2
CD86	IT2.2	^150^Nd	Regulation of T cell activity	CD86

* p-Erk1/2 was omitted from the panel after TNF titration experiments, since TNF stimulation did not alter p-Erk1/2 expression.

** *Metal-conjugation carried out at our laboratory (all other antibodies were purchased pre-conjugated)*.

Thirteen markers were applied to define common PBMC subsets; these were used in both automated clustering and manual gating. Functional markers for TNF signaling were the cleaved caspase 3 as a marker for apoptosis signaling; p-p38 [T180/Y182] and p-Erk1/2 [T202/Y204] as markers for the MAPK-pathway activation; IkBa and p-NFkB p65 [S529] for the NFkB canonical pathway; p-Akt [S473] for the PI3K-Akt pathway; and p-cJun [S73] for the SAPK/JNK signaling pathway. CD86 was added as a marker of T cell regulation and analyzed as functional marker, although signaling through this pathway is not directly related to TNF.

Treatment with PMA resulted in significant increases in Erk1/2 phosphorylation, whereas TNF treatment did not have significant effects on Erk1/2 phosphorylation. This marker was therefore omitted from experiments after panel titration.

### Experimental Workflow

Cryopreserved PBMCs were thawed, rapidly transferred to warm X-VIVO™ containing a nuclease (Benzonase® Nuclease, Merck Millipore, 25 U/mL), followed by centrifugation and resting in X-VIVO™ for 4 h at 37°C, 5% CO_2_. The resting time was optimized in set-up experiments (data not shown). Viability staining was performed according to the manufacturer's instructions with Cell-ID™ cisplatin (Fluidigm). PBMCs from each individual were split into two aliquots; one was not stimulated, and the other was stimulated with 50 ng/mL TNF for 12 min. Stimulation time and dose had been defined after a series of TNF time and dose titrations. Cells in both samples were fixed in proteomic stabilizer (Smart Tubes Inc.) for 10 min and stored at −80°C until barcoding and staining. All cells were barcoded simultaneously with 20-plex Cell-ID™ barcoding kits (Fluidigm) as recommended by the manufacturer. After pooling, surface staining, methanol-permeabilization, and intracellular staining were carried out. PBMCs were then stained with MaxPar DNA intercalator overnight (Fluidigm) and analyzed the following day on a Helios mass cytometer (Fluidigm) after addition of normalization beads (Fluidigm). Raw FCS-files were bead-normalized, concatenated, and debarcoded with software tools from Fluidigm before subsequent analysis.

### Data Analysis Workflow

Three independent analysis pipelines were performed to test and share different approaches as well as to validate our in-house NM2B algorithm (pipeline 1).

#### Pipeline 1: NM2B Algorithm

This algorithm consisted of three main steps: preprocessing (A), finding cell types (B) and classification (C).

- Preprocessing: We fitted a mixture of two Gaussian distributions to mean-variance scaled “Event_length,” “Center,” “Offset,” “Width,” “Residual,” “191Ir_DNA1,” “193Ir_DNA2” markers for cleanup and discarded data belonging to the smaller cluster as doublets and debris (16, 17).
- Finding cell types: We used the following phenotyping markers to detect cell types: “147Sm_CD20,” “170Er_CD3,” “145Nd_CD4,” “146Nd_CD8a,” “169Tm_CD45RA,” “176Yb_CD56,” “148Nd_CD16,” “160Gd_CD14,” “209Bi_CD61,” “159Tb_CD11c,” “151Eu_CD123,” and “174Yb_HLA-DR.” We performed farthest point sampling to find 49 clusters. Farthest point sampling is an approximation to k-means clustering, which can be calculated for large datasets. Clusters of size less than 1/100,000 of the total data size were discarded. We then employed complete linkage meta-clustering of the farthest points with 15 meta-clusters and discarded all meta-clusters of less than 0.5% of the total data size. The final 12 meta-clusters contained a total of 18,374,011 cell events (Supplementary Figure 3).
- Classification: We used the following functional markers as features for classification of individuals as patients or controls: “142Nd_Caspase3,” “156Gd_p-p38,” “152Sm_p-Akt,” “167Er_p-cJun,” “166Er_p-NFkB,” “164Dy_IkBa,” “155Gd_CD120a,” “165Ho_CD120b,” “150Nd_CD86.” For each meta-cluster we calculated the median and 90% quantile of each of the functional markers for all basal cells. In addition, we calculated the arcsinh ratios of the expression of functional markers in stimulated and basal cells. We tested three models, based on either only basal variables (basal), or only arcsinh ratios between stimulated and basal variables (ratio) or both basal and arcsinh ratios (combined). We fitted a logistic lasso regression model, that is a logistic regression model with automatic variable selection, using double leave-one-out cross validation. For details of how double leave-one-out cross-validation was performed we refer to the Supplementary Material. We report cross-validation accuracy, area under the ROC curve (AUC), and all non-zero coefficients.

#### Pipeline 2: CITRUS Algorithm

Normalized, concatenated and debarcoded files were imported in Cytobank for downstream analysis (18). Data were cleaned for doublets, debris and dead cells by biaxial gating and analyzed with the cluster identification, characterization, and regression tool CITRUS in cytobank.org, applying the predictive regression model Nearest Shrunken Centroid/PAMR (19). CITRUS was run on the same data set, but with independent downsampling, with 3 repetitions.

#### Pipeline 3: Manual Analysis

Normalized, concatenated, and debarcoded files were imported in Cytobank. Data were cleaned for doublets, debris, and dead cells by biaxial gating. viSNE analysis based on t-distributed stochastic neighbor embedding was performed for each donor and patient after downsampling to 50,000 cell events per individual and condition (20), and cell subsets were gated on individual viSNE plots (Supplementary Figure 6). Expression of functional markers was compared in all cell subsets, both unstimulated and TNF-stimulated, by applying non-parametric Mann-Whitney U tests using GraphPad prism version 7.0c for Mac OS X. A correction for multiple comparisons was not conducted due to the explorative character of this study.

## Results

### Pipeline 1: NM2B Algorithm

Single-cell data from all 40 individuals were clustered and meta-clustered, and different numbers of clusters and meta-clusters were tested. The model used provided the best translation of meta-clusters into common immune cell subsets. The numbers of clusters and meta-clusters influenced cross-validation accuracy for the classification of RA patients and healthy donors, but when higher numbers of meta-clusters were used, the results were more difficult to interpret with regards to common cell subsets (data not shown).

**Figure 1:**
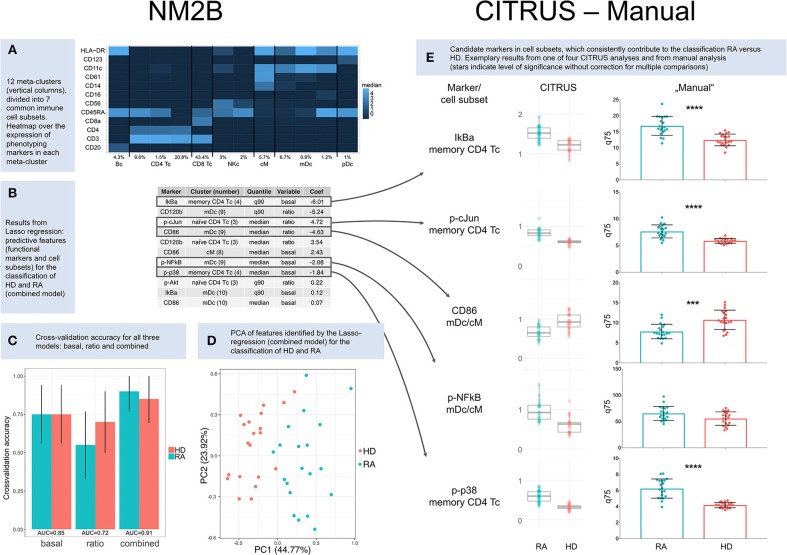
Results. **(A)** Heatmap over the expression of phenotyping markers in 12 meta-clusters (columns). Meta-clusters were identified as B cells (Bc), CD4^+^ T cells (CD4 Tc), CD8^+^ T cells (CD8 Tc), natural killer cells (NKc), classical monocytes (cM), myeloid dendritic cells (mDc), and plasmacytoid dendritic cells (pDc). Relative abundance is given for each cell subset in percent. **(B)** Results from Lasso regression: predictive features (functional markers and cell subsets) and their contribution to the classification of healthy donors (HD) and RA patients (RA). Only nonzero coefficients are shown. Coefficients for CD86 and p-cJun are based on ratios and therefore inverted compared to CITRUS and manual comparisons of basal marker expression. **(C)** Cross-validation accuracy for all three NM2B analyses (“basal,” “ratio,” and “combined”), with area-under-the-curve (AUC) values for ROC analysis. **(D)** Principle component analysis (PCA) of features identified by Lasso-regression (combined model) for the classification of RA patients and healthy donors. **(E)** Cluster identification, characterization, and regression algorithm (CITRUS) and non-parametric testing (Manual). CITRUS results are presented in boxplots, as provided by the algorithm. Results from manual analysis are presented in scatter dot plots. Medians (CITRUS) and 75th percentiles (manual) are plotted for each RA patient (blue) and healthy donor (red); median, and upper and lower quartile. Asterisks indicate level of significance without correction for multiple comparisons (^***^p < 0.001, ^****^p < 0.0001).

Results presented here are based on 49 clusters and 12 meta-clusters; the latter include one B cell meta-cluster (4.3%), four of T cells (75.3%), two of natural killer cells (5%), one of classical monocytes (5.7%), three of myeloid dendritic cells (8.8%) and one of plasmacytoid dendritic cells (1%). Phenotyping markers are differentially expressed in the meta-clusters (Figure 1A); differences in expression of phenotyping markers in healthy donors vs. RA patients were not significant (Supplementary Figure 10).

A regression model based on both basal expression of functional markers and arcsinh ratios (“combined model”) provided the best predictive TNF signaling patterns for healthy donors and RA patients. In this model seven functional markers (IkBa, CD120b, CD86, p-cJun, p-NFkB, p-p38, and p-Akt) in five cell subsets (memory CD4^+^ T cells, CD11c+HLA-DR+CD14^low^CD61^low^ myeloid dendritic cells, naïve CD4^+^CD45RA^+^CD11c^low^ T cells, classical monocytes, and CD11c^high^HLA-DR^high^CD61^low^ myeloid dendritic cells) were identified as predictive markers (Figure 1B). Applying these markers, the combined model correctly classified 18 of 20 RA patients and 17 of 20 healthy donors (Figure 1C). The two patients who were not classified correctly were both females older than 67 years with high disease activity (DAS28 5.3/6.4). One was seronegative and one was being treated with prednisolone at 12.5 mg per day. Principle component analysis (PCA) of features identified by the Lasso-regression showed a good separation of HD vs. RA in the combined (Figure 1D) and basal model, but to a lesser degree in the ratio model (Supplementary Figures 7–9).

### Pipeline 2: CITRUS Algorithm

We performed four repetitive CITRUS analyses of basal expression of functional markers. p-p38, IkBa, p-cJun, p-NFkB, and CD86 were identified as predictive markers by CITRUS, with memory CD4^+^ T cells being the most relevant cell subset for both p-p38, IkBa, and p-cJun, while clusters within myeloid dendritic cell subets (mDc) and classical monocytes (cM) were the most relevant for p-NFkB and CD86 (Supplementary Figure 11 and Supplementary Table 10). There was not always a clear distinction between myeloid dendritic cells and classical monocytes in hierarchical clustering in CITRUS, and both these cell subsets were relevant for the markers p-NFkB and CD86 (Figure 1E and Supplementary Table 10).

Compared to the NM2B and manual analysis, there were slight differences in the weighting of cell subsets for p-cJun (Figure 1E). In four CITRUS analyses, memory CD4^+^ T cells were the primary cell subset of interest for p-cJun, whereas automated analysis pointed to naïve CD4^+^ T cells as the most significant cell subset. In manual analysis, p-cJun expression was significantly different between HD and RA in both naïve and memory CD4 Tc.

Exemplary CITRUS results and cross validation model error rates can be found in Supplementary Figures 11, 12.

### Pipeline 3: Manual Analysis

The manual analysis generally confirmed results from regression data modeling (Figure 1E). For the p-NFkB, regression tools had suggested significant differences in myeloid dendritic cell and classical monocyte subsets. However, in non-parametric testing, p-NFkB expression was not significantly different in myeloid dendritic cell and classical monocyte subsets of healthy donors vs. RA patients, but there were differences in p-NFkB in memory CD4^+^ T cells. Complete results from manual analysis are shown in Supplementary Figures 13–15.

In summary, and based on all three analysis pipelines, we suggest that the phenotyping markers CD4, CD45RA, and CD11c (to identify CD4 naïve and memory CD4^+^ T cells, and myeloid dendritic cells) and the functional markers p-p38, IkBa, p-cJun, p-NFkB, and CD86 may be candidate markers for a simplified setup, e.g., in confirmatory studies by flow cytometry.

## Discussion

In this study, comprehensive investigation of signaling patterns in unstimulated and TNF-stimulated immune cells by mass cytometry revealed cell type specific differences in RA patients compared to healthy donors of the same gender and with similar ages. Applying predictive regression models, we found that the basal expression of p-p38 and IkBa in memory CD4^+^ T cells, p-cJun in naïve, and memory CD4^+^ T cells, and p-NFkB and CD86 in myeloid dendritic cells and classical monocytes differentiated between healthy donors and RA patients. We want to emphasize the explorative character of our study and the role of mass cytometry in this setting. Mass cytometry and related analysis tools are currently not used in routine clinical practice. However, in this study we suggest a smaller set of markers for the distinction between HD and RA. These markers could make our approach applicable and feasible in future research e.g., on a flow cytometry platform. Our data indicate that phenotyping markers CD4, CD45RA, CD11c for the identification of CD4^+^ T cell subsets and myeloid dendritic cells, and p-p38, IkBa, p-cJun, p-NFkB, and CD86 as relevant functional markers could be used to analyze unstimulated PBMCs by flow cytometry for diagnosis and stratification in RA.

Studies of signaling pathways in arthritis are often limited to one or two distinct cell subsets and a few functional markers and are frequently carried out in animal arthritis models. Comprehensive investigation of signaling in immune cell subsets in patients and healthy individuals has been challenging due to technical limitations. High-dimensional mass cytometry can fill a gap as it enables the simultaneous investigation of many markers in millions of heterogeneous cells with a single-cell resolution. Our study utilized a total of 34 channels (including barcoding and beads) and only partially exploited the potential of the technology.

In an analysis of a single RA patient and one healthy donor Nair et al. demonstrated that a complex mass cytometry setup distinguished between health and disease and was able to detect changes after TNF inhibitor treatment (12). Due to the illustrational character of their study, differences in signaling were not quantified, but both p-p38 and p-NFkB were differentiating markers in several cell subsets. Their data pointed to granulocytes as a cell population altered by TNF pathway activation. In support of this, another study had previously shown that granulocytes express high levels of TNF receptors (21). Unfortunately, our study did not include granulocytes as we studied PBMCs. PBMCs were selected to provide a detailed insight into non-granulocyte white blood cell populations, allowing for simultaneous stimulation of cells from the entire cohort under standardized conditions after cryo-preservation.

The use of cryo-preserved PBMCs introduces several potential contributors to variation, and deprives cells from their individual surroundings by the removal of plasma (22–26). We reduced variation through stringent use of standard operating procedures for the handling of live cells from the time of collection to cryo-preservation to resting and stimulation. Moreover, the experimental steps were conducted simultaneously on cells from all donors whenever feasible. However, for future study we would recommend the use of peripheral blood leukocytes with immediate fixation after sample acquisition from patient and donor.

Galligan et al. performed a phospho-flow analysis on PBMCs on a less homogeneous RA population than our cohort. The Galligan et al. cohort included RA patients at different disease stages treated with different medications and patients with osteoarthritis and healthy donors (11). In agreement with our results, they found elevated levels of several phospho-epitopes in CD4^+^ T cell subsets in RA patients compared to healthy donors, and, to a lesser degree, to osteoarthritis patients. Interestingly, there were not significant differences in p38 phosphorylation levels between RA patients and healthy controls in the Galligan et al. study. Unfortunately, markers for the canonical NFkB-signaling pathway were not included.

To identify differences between “healthy” and “sick” representative cohorts of both groups are required. However, the number of simultaneously applicable barcodes, parallel handling of all samples, read-out time on the mass cytometer, and analysis of multi-dimensional data on millions of events set currently limits on cohort sizes. Based on a total of 40 individuals, our study is primarily of explorative character. Our cohorts were sex-matched. We aimed to achieve an age match between patients and healthy donors, although immune status has been shown to be rather stable over time in healthy adults (27). Our experimental setup allowed for a high degree of simultaneous analysis, running 80 samples (20HD+20RA in two conditions) at the same time. For future studies with more samples, it is important to assure that results are robust across different cytometry runs, e.g., through the use of a reference sample.

We only included newly diagnosed patients, in whom disease-related immune status was unaffected by immune-modulatory or immune-suppressive treatment with the exception of low-to-moderate dosages of prednisolone in five of the 20 patients. While prednisolone treatment may introduce an unwanted heterogeneity, this reflects the real-life situation at rheumatology outpatient clinics, with some patients being referred after pre-treatment. In a sub-group analysis with CITRUS we couldn't identify factors that differentiated prednisolone-treated from prednisolone-naïve patients.

RA is an inflammatory condition, and untreated patients are expected to express signs of inflammation on a cellular level compared to healthy donors. In our RA cohort, 15 patients had elevated levels of CRP, including all five patients treated with prednisolone. The higher levels of markers known to be involved in inflammatory signaling pathways, such as the canonical NFkB and the MAPK signaling pathways, in patients compared to healthy donors in this cohort was, therefore, not a surprise. We did not include patients with different inflammatory conditions in our study, hence the specificity of the observed signaling signatures for RA compared to other inflammatory conditions is not known. For future studies, cohorts with other TNF-driven conditions such as e.g., inflammatory bowel diseases should be added as disease controls.

That CD86 was consistently expressed to a lesser degree on classical monocytes and myeloid dendritic cells of RA patients compared to healthy donors is likely relevant to the pathogenesis of RA. CD86 is highly expressed on antigen-presenting cells in synovial fluid and synovia of RA patients, whereas CD28, the T cell counterpart of CD86, is expressed at lower levels in patients with active RA compared to healthy donors (28).

In conclusion, this study provided insight into TNF-mediated signaling patterns, which are distinct for RA patients compared to healthy individuals. A comprehensive understanding of signaling signatures may facilitate more accurate diagnosis, better stratification of patients to guide treatment decisions, and the identification of candidate treatment targets in RA patients.

## Data Availability

The datasets for this study (normalized, concatenated, debarcoded, and after removal of events compromised by injector clogging can be found in the Flow Repository https://flowrepository.org/, experiment-ID FR-FCM-Z24N.

## Ethics Statement

All donors and patients gave written informed consent for inclusion into the Norwegian Arthritis Registry (NorArtritt) and the Research Biobank for Rheumatic Diseases in Western Norway (approval REK 2012/1689). Utilization of registry data and biobank material for this study was approved by the Regional Ethics Committee (approval REK 2014/317).

## Author's Note

A summary of this work was presented in poster form at Cyto2018 in Prague, abstract B13 156.

## Author Contributions

All authors have contributed to manuscript review. LB has contributed with experiment design, collection of samples and clinical data, cytokine and antibody titration, all laboratory work, data analysis pipeline 1 and 2, manuscript writing. S-EG has contributed to cytokine and antibody titrations, followed critically through especially the data analysis part of this study. NB and MB have contributed with data analysis pipeline 3 and manuscript writing. GB has contributed with laboratory work, ciritical follow-up through all stages of this study. AS has contributed with initial experiments for TNF stimulation and background work on TNF receptors/TNF receptor antibodies. CG has contributed to experiment design and facilitated collection of samples and clinical data. CV has contributed to experiment design and facilitated sample collection, laboratory work, and mass cytometry experiments. SG has contributed to experiment design and critical supervision throughout all parts of this study.

### Conflict of Interest Statement

The authors declare that the research was conducted in the absence of any commercial or financial relationships that could be construed as a potential conflict of interest.
