# Supplementary material for: Candidate Markers for Stratification and Classification in Rheumatoid Arthritis
Source: Front Immunol. 2019 Jul 5;10:1488. doi: 10.3389/fimmu.2019.01488 (PMC6626904; doi:10.3389/fimmu.2019.01488)
Supplement: Supplementary file 1 [file Data_Sheet_1.pdf]

Supplementary Material: Candidate markers for stratification and classification in rheumatoid arthritis

INTRODUCTION

Information essential for the understanding of the main results of our work is presented in the main article. In-depth information on material, methods and results is provided here in the supplementary materials *in the same order and with the same headings/sub-headings as in the main article*.

MATERIALS AND METHODS

Peripheral blood mononuclear cells (PBMCs)

The following protocol applies to the collection of *all* PBMCs used in this study (healthy donors, patients, PBMCs for TNF titrations, panel titration, main experiments).

Biobank nurse at the Rheumatology department:

- After taking samples from the patient in 4 CPT, turn the tubes immediately and gently x 10. Proceed to centrifugation within max. 2 hours
- Turn the CPT gently x 5 before centrifugation
- Spin: acceleration 9, deceleration 9, 1800 G, 35 min, 22 °C
- Visual control after centrifugation, all RBC should have migrated through the gel and you should see the "milky" layer of PBMC on top of the gel
- Turn the CPT gently x 5

Biotechnician at the laboratory:

- Pick up CPT. Prepare the cell.lab, switch on the hood, warm up PBS to room temperature (RT)
- Turn the CPT gently x 5
- Remove all plasma and cells and pipet into a sterile 50 mL Falcon
- Add PBS at RT up to 50 mL and turn gently x 5
- Spin: acc. 9, dec. 9, 300 G, 15 min, 4 °C
- Remove supernatant but 1-2 mL, don't remove pellet
- Dissolve the pellet carefully, pipette not smaller than 1 mL
- Add PBS up to 30 mL and turn gently x 5
- Spin: acc. 9, dec. 9, 300 G, 10 min, 4 °C
- While spinning: prepare ice, cryo tubes, DMSO, X-vivo (cold), ProFreeze (cold), Countess, Trypan blue, CoolCell® freezing containers should now stand in the fridge.
- Fill 180 µL X-vivo into an Eppendorff tube
- After centrifugation: remove supernatant, but not cell pellet
- Carefully dissolve the pellet in 2 mL X-vivo (concentration 1)

- Transfer 20 µL concentration 1 into your Eppendorf tube with 180 µL X-vivo (1:10, concentration 2)
- Mix 20 µL of your concentration 2 with 20 µL Trypan blue (1:2, concentration 3).
- Fill the reading chamber of the Countess with concentration 3, 10 µL
- Do a cell count. Dilute concentration 1 to a final concentration of about 20 x 10^6 PBMC/mL.
- The final cell suspension consists of PBMC, 50% X-vivo, 42.5% ProFreeze and 7.5% DMSO.
- Add X-vivo to concentration 1 so that cell suspension volume is 50% of the total planned volume
- In a new tube you mix the other half of the total planned volume, containing 85% ProFreeze and 15% DMSO
- Set cryo vials on ice
- From this step on you want to work quickly, no breaks: gently add the ProFreeze/DMSO-solution to the cell/X-vivo solution and mix by shaking the tube gently
- Pipet 1 mL/tube cell+DMSO solution rapidly into cryo vials
- Close vials, set vials into freezing containers, and freeze at -80 °C. Empty holes in the freezing containers have to be filled with dummies

Antibody panel

Antibodies

Antibodies were purchased pre-conjugated except the CD120a-, CD120b- and p-cJun-antibodies, which were conjugated at our laboratory applying Fluidigm Maxpar antibody labeling kits and protocols: https://www.fluidigm.com/binaries/content/documents/fluidigm/resources/maxpar-antibody-labeling-kit-pr-prd002/maxpar-antibody-labeling-kit-pr-prd002/fluidigm%3Afile

Tumor necrosis factor (TNF) dose and time titration

To define optimal dosing and stimulation time for TNF, we performed a TNF dose and time titration on PBMC from two healthy donors with the following panel, setup and protocol:

Barcode	purpose	contents
1	dose (time 15 min)	unstim
2	"	6.25 ng
3	"	12.5 ng
4	"	25 ng
5	"	50 ng
6	"	100 ng
7	time (dose 50 ng)	3'45''
8	"	7'30''
9	"	15'
10	"	30'
11	"	60'
12	"	120'

Supplementary table 1: Experiment design for TNF dose and time titrations.

Metal	Target	Clone	Dilution	Amount x1	Amount x2
			conc.	mcL	mcL
89Y	**CD45**	HI30	800x	0,125	0,25
145Nd	**CD4**	RPA-T4	800x	0,125	0,25
146Nd	**CD8a**	RPA-T8	400x	0,25	0,5
147Sm	**CD20**	2H7	400x	0,25	0,5
148Nd	**CD16**	3G8	400x	0,25	0,5
150Nd	**CD86**	IT2.2	800x	0,125	0,25
151Eu	**CD123 (IL-3R)**	6H6	800x	0,125	0,25
155Gd	**CD120a**	MABTNFR1-B1	100x	2	4
159Tb	**CD11c**	Bu15	800x	0,125	0,25
160Gd	**CD14**	M5E2	200x	0,5	1
163Dy	**CD56 (NCAM)**	NCAM16.2	1600x	0,063	0,125
165Ho	**CD120b**	hTNFR-M1	100x	2	4
169Tm	**CD45RA**	HI100	1600x	0,063	0,125
170Er	**CD3**	UCHT1	800x	0,125	0,25
174Yb	**HLA-DR**	L243	800x	0,125	0,25
209Bi	**CD61**	VI-PL2	1600x	0,063	0,125
			Sum antib.	6,314	12,625
			CSB	43,686	87,375
			Mastermix	50	100
			Staining vol.	100	200
142Nd	**Caspase 3 (Cleaved)**	D3E9	200x	0,5	1
152Sm	**pAkt [S473]**	D9E	100x	1	2
156Gd	**p-p38 [T180/Y182]**	D3F9	200x	0,5	1
164Dy	**IkBa**	L35A5	200x	0,5	1
166Er	**pNF-kB p65 [S529]**	K10-895.12.50	400x	0,25	0,5
167Er	**p-cJun [s73]**	D47G9	100x	2	4
171Yb	**pERK 1/2 [T202/Y204]**	D13.14.4E	100x	1	2
			Sum antib.	5,75	11,5
			CSB	44,25	88,5
			Mastermix	50	100
			Staining vol.	100	200

Supplementary table 2: Antibody panel for TNF dose and time titrations.

**Day 1 Preparations, thawing, resting**	◻	1. Prepare warm X-vivo (37 °C, in the following referred to as body temperature, BT) in 2 x 4 mL Falcon tubes enriched with 1 mcL/250 U Benzonase (≈ 36 U/mL)
	◻	2. Set centrifuge to room temperature (RT)
	◻	3. Take out 3 cryo-tubes with cells from both healthy donors (HD), quick-thaw cells in warm X-vivo, spin 300 G/7 min
	◻	4. Resuspend in 5 mL/tube X-vivo and do a cell count
	◻	5. Adjust cell numbers, aim at 6-10 x 10^6 per HD
	◻	6. Fill up with X-vivo to a total volume of 12 mL/tube
	◻	7. Set cells to rest in incubator 4h, mix cells carefully and wash off the walls twice within and once at the end of incubation
	◻	8. Thaw TNF 1000 ng/mL, 1 vial and prepare titration steps
**Dead cell stain**	◻	9. Set Maxpar Cell Staining Buffer 25 mL and PBS 2 mL to BT
	◻	10. Spin cells at 300G/7 min
	◻	11. Prepare PBS 2 mL + 2.22 mcL cisplatin
	◻	12. Resuspend pellets in 1 mL PBS/cisplatin mix per tube
	◻	13. Mix well and incubate at RT for max. 5 minutes
	◻	14. Add warm Maxpar Cell Staining Buffer 12 mL per tube to quench cisplatin
**Stimulation, fixation**	◻	15. Centrifuge cells 300 G/7 min and discard supernatant
	◻	16. Resuspend in 3.5 mL X-vivo
	◻	17. Divide: 0.25 mL x 12 in FACS tubes
	◻	18. Unstim: add 0.25 mL X-vivo
	◻	19. Stim: add TNF in titration concentrations. Fixed amount of TNF 50 ng for time titration
	◻	20. Fix all cells in proteomic stabilizer (add 0.7 mL proteomic stabilizer to 0.5 mL cell suspension)
	◻	21. Mix gently and thoroughly, incubate for 10 min at RT
	◻	22. Freeze all tubes at -80
**Day 2: Barcoding**	◻	23. Add cold Maxpar PBS to frozen cell suspension and thaw slowly. When completely thawed, spin 800G, 5 min
	◻	24. Prepare Maxpar Barcode Perm Buffer (BPB), 65 mL (6,5 mL Maxpar BPB 10x plus 58.5 mL Maxpar PBS)
	◻	25. Discard supernatant, resuspend in 1 mL BPB, spin and repeat for another wash
	◻	26. Prepare barcodes 10 mcL/barcode in 100 mcL BPB
	◻	27. Resuspend pellets in 400 mcL BPB, add barcodes 50 mcL, mix and incubate for 30 min at RT on a shaker
	◻	28. Wash twice in 1 mL Maxpar CSB, resuspend in 100 mcL CSB, and unite all samples from one HD in one tube
	◻	29. Add CSB to 3 mL, do a cell count and spin 800G/5 min
**Surface stain**	◻	30. Discard supernatant, adjust to a staining volume of 100 mcL
	◻	31. Add 100 mcL of surface antibody so the total staining volume is 200 mcL
	◻	32. Gently vortex sample and incubate for 30 minutes, RT, shaker

	□	33. Wash by adding 1 mL Maxpar CSB, centrifuge, discard supernatant
**Permeabili-zation**	□	34. Resuspend in residual volume and chill samples on ice for 10 minutes
	□	35. Add ice-cold MeOH 0.5 mL and mix gently
	□	36. Incubate for 15 minutes on ice
	□	37. Wash cells with 1 mL Maxpar Cell Staining Buffer, centrifuge and discard supernatant by aspiration. Repeat for a total of 2 washes
**Signaling stain**	□	38. Adjust cell suspension volume to 100 mcL
	□	39. Add 100 mcL of signaling antibody cocktail so the total staining volume is 200 mcL
	□	40. Gently vortex sample and incubate for 30 minutes, RT, shaker
	□	41. Wash by adding 1 mL Maxpar Cell Staining Buffer, centrifuge, discard supernatant
	□	42. Repeat for a total of 2 washes
**Intercalation**	□	43. Add 1 mL of intercalation solution per tube and gently vortex. Incubate overnight in fridge
**Day 3: Data acquisition**	□	44. Wash cells by adding 4 mL of Maxpar Cell Staining Buffer, centrifuge and discard supernatant
	□	45. Repeat for a total of 2 washes with Maxpar Cell Staining Buffer
	□	46. Wash cells with 4 mL of Maxpar Water, centrifuge and discard supernatant
	□	47. Leave cells pelleted until ready to run mass cytometry. Immediately prior to analysis, adjust cell concentration to 5 x 10^5/mL with Maxpar Water+Beads and filter cells into cell strainer cap tubes
	□	48. Acquire data on Helios

Supplementary table 3: Protocol for TNF dose and time titrations.

Panel titration

Antibody titration was carried out on PBMCs from one healthy donor. The antibodies were divided into three subpanels to keep open channels potentially receiving signal spillover. Known sources of signal spillover in mass cytometry are direct spillover into neighboring channels (metal mass ±1 Da), spillover due to isotope oxidation (into the +16 Da channel, for example from 150Nd to 166Er) and metal impurities (for example spillover from a Nd-isotope into other channels of other Nd-isotopes). For the titration of signaling markers, PBMC were stimulated simultaneously with TNF 50 ng/mL and PMA 100 nM/mL. Retrospectively, and for the purpose of this antibody panel with regards to the main experiment (chapter 2.4), PBMC should favorably have been stimulated with TNF only and a backbone panel consisting of central phenotyping markers should have been applied to each titration sub-panel (Gullaksen SE, Bader L, Hellesoy M, Sulen A, Fagerholt OHE, Engen CB, et al. Titrating Complex Mass Cytometry Panels. Cytometry A. 2019).

Panel 1 surface	Panel 1 signaling
• CD16 148Nd<br>• CD123 151Eu<br>• CD120a 155Gd<br>• CD11c 159Tb<br>• CD45RA 169Tm<br>• CD56 176Yb	• Caspase 3 142Nd<br>• p-NFkB 166Er
**Panel 2 surface**	**Panel 2 signaling**
• CD4 145Nd<br>• CD20 147Sm<br>• CD86 150Nd	• p-Akt 152Sm<br>• p-p38 156Gd<br>• IkBa 164Dy<br>• p-cJun 167Er<br>• p-Erk1/2 171Yb
**Panel 3 surface**	**Panel 3 signaling**
• CD45 89Y<br>• CD8a 146Nd<br>• CD14 160Gd<br>• CD120b 165Ho<br>• CD3 170Er<br>• HLA-DR 174Yb<br>• CD61 209Bi	

Supplementary table 4: Sub-panels for antibody titrations.

For each sub-panel:		
Tube	Barcode 1 unstim	Barcode 2 stim
1	100x	100x
2	200x	200x
3	400x	400x
4	800x	800x
5	1600x	1600x

Supplementary table 5: Experiment design for panel titration.

**Day 1 Preparations, thawing, resting**	□	1. Prepare warm X-vivo (BT) in a 50 mL Falcon tube, 40 mL
	□	2. Set centrifuge to RT
	□	3. Take out 9 cryo-tubes with cells KTR-017, quick-thaw cells in warm X-vivo, spin 300 G/15 min
	□	4. Resuspend in 20 mL X-vivo and do a cell count
	□	5. Fill up with X-vivo to a total volume of 30 mL
	□	6. Set cells to rest in incubator 4h, mix cells carefully and wash off the walls twice within and once at the end of incubation
	□	7. Do another cell count at the end of incubation
	□	8. Take out TNF 200 ng/mL, 1 vial and PMA for a final concentration of 100 nM/mL
	□	9. Set Maxpar Cell Staining Buffer 45 mL and PBS 5 mL to BT
	□	10. Spin cells at 300G/10 min
	□	11. Prepare PBS 4 mL + 4.44 mcL cisplatin
**Dead cell stain**	□	12. Resuspend pellet in 4 mL PBS/cisplatin mix
	□	13. Mix well and incubate at RT for max. 5 minutes
	□	14. Add warm Maxpar Cell Staining Buffer 45 mL to quench cisplatin
	□	15. Centrifuge cells 300 G/15 min and discard supernatant
	□	16. Resuspend in 6.1 mL X-vivo
**Stimulation, fixation**	□	17. Divide: 3 mL x 2 in 2 Falcon tubes
	□	18. Unstim: add 1 mL X-vivo
	□	19. Stim: TNF 200 ng/mL + PMA for 13 min (add 1 mL of TNF/PMA mix), final concentrations TNF 50 ng/mL, PMA 100 nM/mL
	□	20. Fix all cells in proteomic stabilizer (add 5.6 mL proteomic stabilizer to 4 mL cell suspension)
	□	21. Mix gently and thoroughly, incubate for 10 min at RT
	□	22. Freeze all tubes at -80
**Day 2: Barcoding**	□	23. Add cold Maxpar PBS to frozen cell suspension and thaw slowly. When completely thawed, spin 800G, 7 min
	□	24. Prepare Maxpar Barcode Perm Buffer (BPB), 20 mL (2 mL Maxpar BPB 10x plus 18 mL Maxpar PBS)
	□	25. Discard supernatant, resuspend in 4 mL BPB, spin and repeat for another wash
	□	26. Prepare barcodes 20 mcL/barcode in 200 mcL BPB
	□	27. Resuspend pellets in 1.6 mL BPB, add barcodes, mix and incubate for 30 min at RT on a shaker
**Surface stain**	□	28. Wash twice in 4 mL Maxpar CSB, resuspend in 200 mcL CSB, mix unstim and stim in 1 tube, add 400 mcL Maxpar CSB and aliquot 50 mcL x 15 (panel 1, 2, 3 x 5) in a 96-deep-well plate

	☐	29. Add 50 mcL of surface antibody so the total staining volume is 100 mcL
	☐	30. Gently vortex sample and incubate for 30 minutes, RT, shaker
	☐	31. Wash by adding 1 mL Maxpar CSB, centrifuge, discard supernatant
**Permeabili-zation**	☐	32. Resuspend in residual volume and chill samples on ice for 10 minutes
	☐	33. Add ice-cold MeOH 0.5 mL and mix gently
	☐	34. Incubate for 15 minutes on ice
	☐	35. Wash cells with 1 mL Maxpar Cell Staining Buffer, centrifuge and discard supernatant by aspiration. Repeat for a total of 2 washes
**Signaling stain**	☐	36. Adjust cell suspension volume to 100 mcL
	☐	37. Add 100 mcL of signaling antibody cocktail so the total staining volume is 200 mcL
	☐	38. Gently vortex sample and incubate for 30 minutes, RT, shaker
	☐	39. Wash by adding 2 mL Maxpar Cell Staining Buffer, centrifuge, discard supernatant
	☐	40. Repeat for a total of 2 washes
**Intercalation**	☐	41. Add 1 mL of intercalation solution per tube and gently vortex. Incubate overnight in fridge
**Day 3: Data acquisition**	☐	42. Wash cells by adding 10 ml of Maxpar Cell Staining Buffer, centrifuge and discard supernatant
	☐	43. Repeat for a total of 2 washes with Maxpar Cell Staining Buffer
	☐	44. Wash cells with 10 ml of Maxpar Water, centrifuge and discard supernatant
	☐	45. Leave cells pelleted until ready to run mass cytometry. Immediately prior to analysis, adjust cell concentration to 5 x 10^5/mL with Maxpar Water+Beads and filter cells into cell strainer cap tubes
	☐	46. Acquire data on Helios

Supplementary table 6: Protocol for antibody panel titrations.

.Fcs-files for each titration step (100x, 200x, 400x, 800x, 1600x) were downsampled to the same cell number and concatenated side-by-side in FlowJo in decreasing antibody concentration. For the final panel, antibody concentrations were chosen which optimzed the ability of the antibody to discern positive from negative populations (for markers with a bimodal distribution) or resulted in the highest ratio stimulated/unstimulated (for functional markers) against the amount of signal spillover into other channels. We calculated antibody signal and tolerance, following recommendations from Fluidigm: https://www.fluidigm.com/binaries/content/documents/fluidigm/search/hippo%3Aresultset/maxpar-panel-designer/fluidigm%3Afile

A

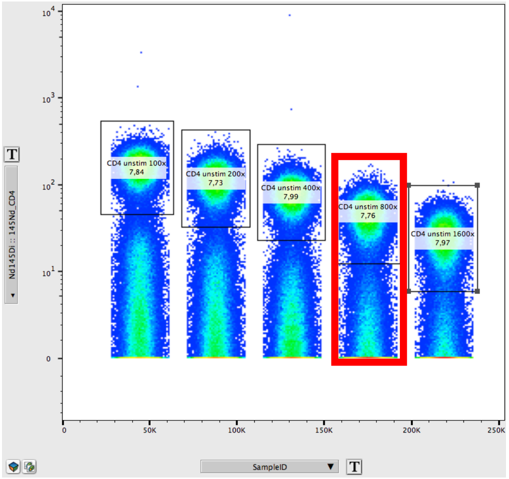

75th percentile

Purpose	Target	Metal	100x	200x	400x	Fluidigm	800x	1600x
Nd	Caspase3	142Nd	0	0	0		0	0
	CD4	145Nd	193	138	93,3	70	57	31,5
+1, Nd	CD8a	146Nd	2,91	1,55	1,34		0	0
Nd	CD16	148Nd	0	0	0		0	0
	Tolerance		38,6	27,6	18,66	14	11,4	6,3

B

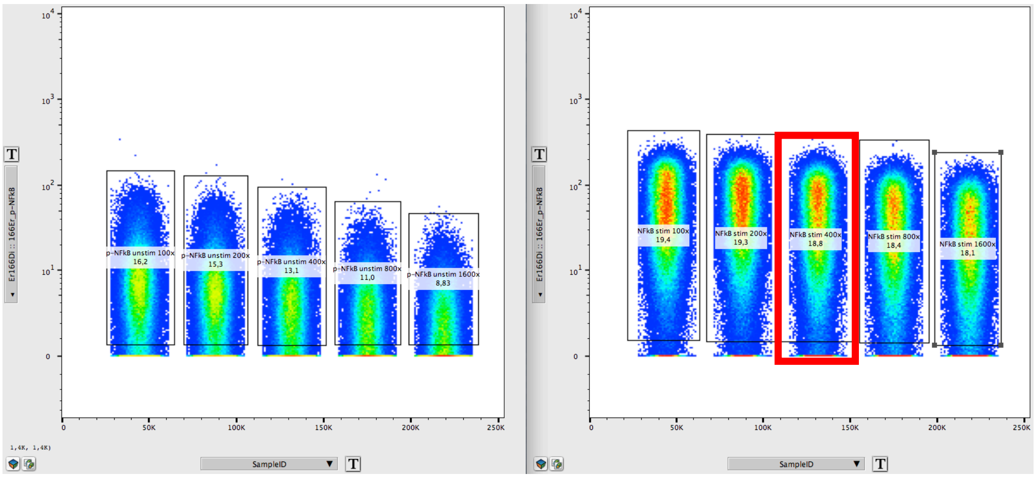

75th percentile

Purpose	Target	Metal	100x	200x	400x	Fluidigm	800x	1600x
-1	CD120a	165Ho	0	0	0		0	0
	p-NFκB uns	166Er	11,6	9,33	7,46	7	6,16	5,14
	p-NFκB stim	166Er	84,4	83,7	70,3		55,9	43,5
	Ratio		7,275862	8,971061	9,423592		9,074675	8,463035
+1/Er	p-cJun	167Er	0,2	0,18	0		0	0
	Tolerance		2,32	1,866	1,492	1	1,232	1,028

Supplementary Figure 1. Antibody titration exemplified on the phenotyping marker CD4 145Nd (A) and the functional marker p-NFkB 166Er. Concatenated files and tables listing 75th percentiles of dual count expression for each antibody concentration (100x, 200x, 400x, 800x, 1600x). The tables list also channels of potential spillover. Red boxes indicate chosen antibody concentrations. As a reference, marker expression and tolerance as measured by the vendor Fluidigm are listed (green background).

Experimental workflow

The main experiment was carried out on three "experiment days". Day 1 consisted of 10 donor/patient samples in 2 conditions (unstimulated/TNF-stimulated) and was carried out 4 times to cover all donors/patients (day 1A, 1B, 1C, 1D). Day 2 and 3 were performed for all 80 samples ((20 donors + 20 patients) x 2 conditions) simultaneously.

Metal	Target	Clone	Dilution	Amount x1	Amount x5
					x5
			conc.	mcL	mcL
89Y	**CD45**	HI30	800x	0,125	0,625
145Nd	**CD4**	RPA-T4	800x	0,125	0,625
146Nd	**CD8a**	RPA-T8	400x	0,25	1,25
147Sm	**CD20**	2H7	400x	0,25	1,25
148Nd	**CD16**	3G8	400x	0,25	1,25
150Nd	**CD86**	IT2.2	800x	0,125	0,625
151Eu	**CD123 (IL-3R)**	6H6	800x	0,125	0,625
155Gd	**CD120a**	MABTNFR1-B1	100x	2	10
159Tb	**CD11c**	Bu15	800x	0,125	0,625
160Gd	**CD14**	M5E2	200x	0,5	2,5
163Dy	**CD56 (NCAM)**	NCAM16.2	1600x	0,063	0,315
165Ho	**CD120b**	hTNFR-M1	100x	2	10
169Tm	**CD45RA**	HI100	1600x	0,063	0,315
170Er	**CD3**	UCHT1	800x	0,125	0,625
174Yb	**HLA-DR**	L243	800x	0,125	0,625
209Bi	**CD61**	VI-PL2	1600x	0,063	0,315
			Sum antib.	6,314	31,57
			CSB	43,686	218,43
			Mastermix	50	250
			Staining vol.	100	100*5
142Nd	**Caspase 3 (Cleaved)**	D3E9	200x	0,5	2,5
152Sm	**pAkt [S473]**	D9E	100x	1	5
156Gd	**p-p38 [T180/Y182]**	D3F9	200x	0,5	2,5
164Dy	**IkBa**	L35A5	200x	0,5	2,5
166Er	**pNF-kB p65 [S529]**	K10-895.12.50	400x	0,25	1,25
167Er	**p-cJun [s73]**	D47G9	100x	2	10
171Yb	**pERK 1/2 [T202/Y204]**	D13.14.4E	100x	1	5
			Sum antib.	5,75	28,75
			CSB	44,25	221,25
			Mastermix	50	250
			Staining vol.	100	100*5

Supplementary table 7: Antibody panel for the main experiment.

**Day 1 Preparations, thawing, resting**	□	1. Prepare 10 Falcon tubes filled with 5 mL X-vivo (BT), enriched with Benzonase for a final concentration of ca 25 U/mL (60 mL X-vivo + 8 mcL Benzonase)
	□	2. Set centrifuge to RT
	□	3. Take out 2 cryo-tubes with cells/donor, quick-thaw cells in warm X-vivo, spin 300 G/7 min
	□	4. Resuspend in 5 mL X-vivo and do a cell count, keep a fixed number of cells in 5 mL X-vivo, e.g. 5×10^6
	□	5. Set cells to rest in incubator 4h, mix cells carefully and wash off the walls once within and once at the end of incubation
	□	6. Thaw pre-prepared cryo-tubes unstim/stim, BT
	□	7. Set Maxpar Cell Staining Buffer 55 mL and PBS 7 mL to BT
	□	8. Spin cells at 300G/5 min
**Dead cell stain**	□	9. Prepare PBS 6 mL + 6.66 mcL cisplatin
	□	10. Resuspend each pellet in 0.5 mL PBS/cisplatin mix
	□	11. Mix well and incubate at RT for max. 5 minutes
	□	12. Add warm Maxpar Cell Staining Buffer 5 mL to quench cisplatin
	□	13. Spin cells 300 G/7 min
	□	14. Discard supernatant and resuspend each pellet in 510 mcL X-vivo
**Stimulation, fixation**	□	15. Divide: 250 mcL x 2 unstim/stim
	□	16. Incubate at RT for 12 minutes
	□	17. Fix all cells in proteomic stabilizer (add 0.7 mL stabilizer to 0.5 mL cell suspension)
	□	18. Mix gently and thoroughly, incubate for 10 min at RT
	□	19. Freeze all tubes at -80 °C
**Day 2: Barcoding**	□	20. Add cold Maxpar PBS 2.4 mL to frozen cell suspension and thaw slowly. When completely thawed, spin 800G, 5 min
	□	21. Prepare Maxpar Barcode Perm Buffer (BPB), 320 mL (32 mL Maxpar BPB 10x plus 288 mL Maxpar PBS)
	□	22. Discard supernatant, resuspend in 1 mL BPB, transfer to 96-deepwell-plate and repeat for another wash with 1 mL BPB
	□	23. Further steps on ice
	□	24. Prepare barcodes: resuspend 2 complete strips (BC 1-20) in 100 mcL BPB and transfer to a 96-well plate
	□	25. Discard supernatant. Further work with multipipette
	□	26. Resuspend pellets in 300 mcL BPB, and add barcodes 50 mcL/well, mix and incubate for 30 min at RT on vortex
	□	27. Wash twice with 1.5 mL Maxpar CSB, resuspend each sample in 200 mcL CSB, and combine all samples of one set of barcodes in one Falcon tube (Tube 1A, 1B, 1C, 1D)
	□	28. Add CSB up to 10 mL and spin 7 min/800 G
**Surface stain**	□	29. Discard supernatant, adjust volume to 50 mcL
	□	30. Add 50 mcL of surface antibody so the total staining volume is 100 mcL
	□	31. Gently vortex sample and incubate for 30 minutes, RT, shaker

	□	32. Wash by adding 4 mL Maxpar CSB, spin, discard supernatant
**Permeabili-zation**	□	33. Resuspend in residual volume and chill samples on ice for 10 minutes
	□	34. Add ice-cold MeOH 1 mL and mix gently
	□	35. Incubate for 15 minutes on ice
	□	36. Wash cells with 4 mL Maxpar Cell Staining Buffer, centrifuge and discard supernatant by aspiration. Repeat for a total of 2 washes
**Signaling stain**	□	37. Adjust cell suspension volume to 50 mcL
	□	38. Add 50 mcL of signaling antibody cocktail so the total staining volume is 100 mcL
	□	39. Gently vortex sample and incubate for 30 minutes, RT, shaker
	□	40. Wash by adding 4 mL Maxpar Cell Staining Buffer, spin, discard supernatant
	□	41. Repeat for a total of 2 washes
**Intercalation**	□	42. Add 1 mL of intercalation solution per tube and gently vortex. Incubate overnight in fridge
**Day 3: Data acquisition**	□	43. Wash cells by adding 10 ml of Maxpar Cell Staining Buffer, centrifuge and discard supernatant
	□	44. Repeat for a total of 2 washes with Maxpar Cell Staining Buffer
	□	45. Wash cells with 10 ml of Maxpar Water, centrifuge and discard supernatant
	□	46. Leave cells pelleted until ready to run mass cytometry. Immediately prior to analysis, adjust cell concentration to 5 x 10^5/mL with Maxpar Water+Beads and filter cells into cell strainer cap tubes
	□	47. Acquire data on Helios

Supplementary table 8: Protocol for the main experiment.

Data analysis workflow

Pipeline 1: NM2B algorithm

The script for this algorithm is available upon request to the corresponding author.

Our approach for preprocessing was inspired by a talk by Bruce Bagwell [Bagwell. A New Analytic Approach for Live Singlet Identification], where he described how to use "Event_length", "Center", "Offset", "Width", "Residual", "191Ir_DNA1", "193Ir_DNA2" markers for identifying live singlets. Bagwell gated manually on a t-SNE plot for identifying cell populations. We decided on a different approach, where we cluster cells based on these markers directly in the 7-dimensional space, since clustering on a t-SNE plot is in general not recommended. We first used mean-variance scaling for each of the markers in order to get comparable features. We then fit a Gaussian mixture model of two multidimensional Gaussian distributions by expectation maximization and used it for clustering. The Gaussian mixture model was fit using the `Mclust` function from the `mclust` package.

The Gaussian mixture model resulted in a total of 94.2% of cells classified as live singlets and analyzed further. The remaining 5.8% of cells were discarded as doublets and debris.

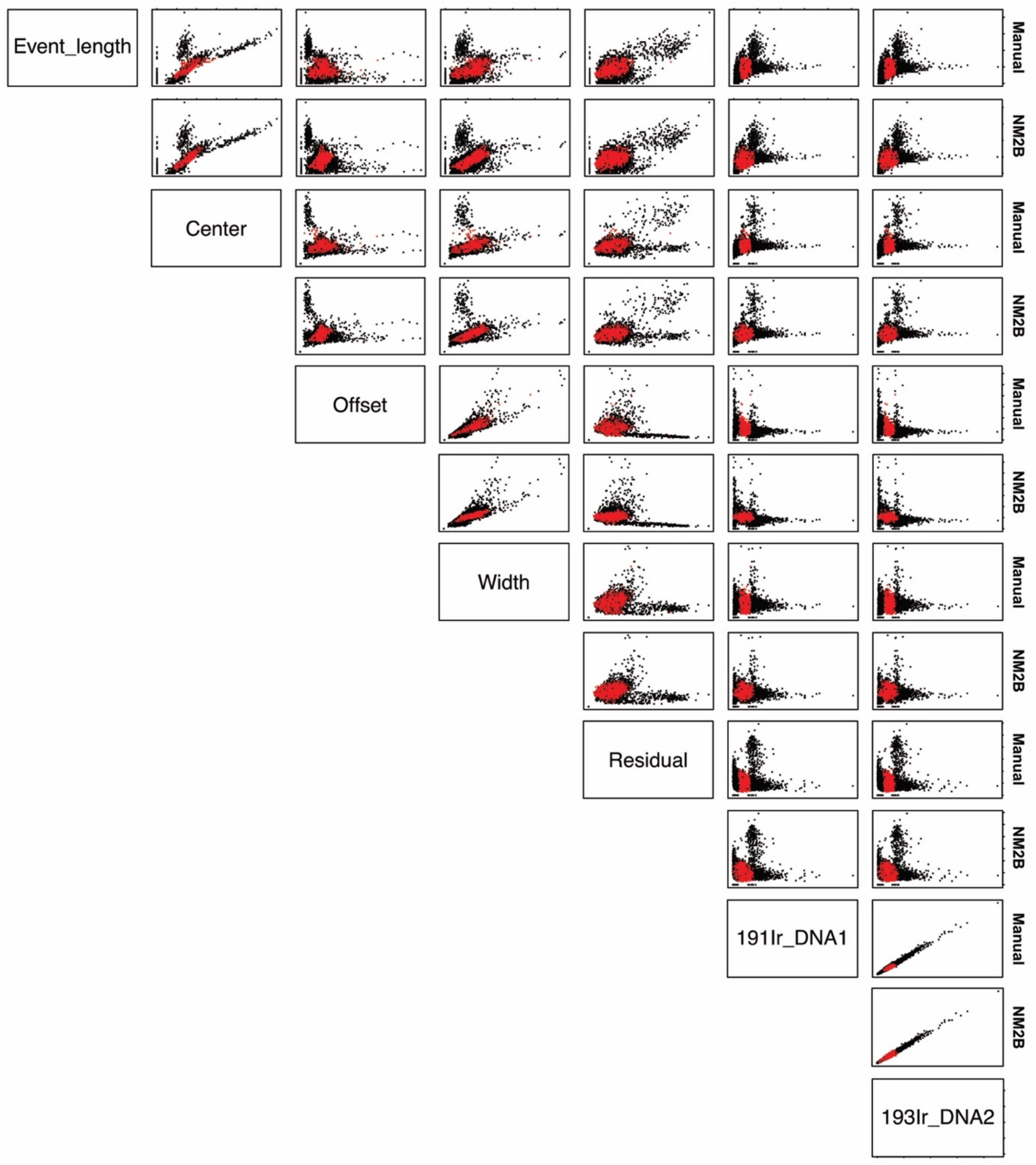

Supplementary figure 2: Overlay of all events (black) and single cells (red) identified by manual and NM2B clean-up. Note that plots are not density plots, ≥88% of cells lie within each red field.

	NM2B		
Manual		Debris/doublets	Single cells
	Debris/doublets	**3.88**	**5.46**
	Single cells	**1.98**	**88.68**

Supplementary table 9: Agreement plot for manual versus NM2B data clean-up in percentages. Both approaches agreed on 3.88% of events in the category “debris/doublets” and 88.68% of events in the category “single cells”.

Our approach for finding cell types relied on splitting the dataset up into many small clusters and then using combining such small homogeneous clusters into meta-clusters. We used the following phenotyping markers to detect cell types: "147Sm_CD20", "170Er_CD3", "145Nd_CD4", "146Nd_CD8a", "169Tm_CD45RA", "176Yb_CD56", "148Nd_CD16", "160Gd_CD14", "209Bi_CD61", "159Tb_CD11c", "151Eu_CD123", "174Yb_HLA-DR". All data was arcsinh-transformed with a cofactor of 5 as suggested by [Qui P., "Toward deterministic and semiautomated SPADE analysis.," Cytometry A, vol. 91, issue 3, pp. 281-289, 2017.]. Farthest-point sampling is a fast algorithm to cluster cells by beginning with an initial point and then until one has the desired number of clusters selecting the point that is farthest away from the closest of all previously selected points. These selected points are then considered as the cluster centers and all remaining points are assigned to the closest cluster center. This procedure can be shown to approximate k-means clustering. When splitting the data into enough clusters this way, this results in relatively small homogeneous clusters. We discarded all clusters of size less than 1e-5 of the total data size. We then used complete-linkage meta-clustering of the cluster-centers. Finally, we discarded all meta-clusters of less than 5e-3 of the total data size. We used farthest-point sampling with 49 clusters and meta-clustering with 15 meta-clusters. We also tested other number of clusters, meta-clusters and thresholds for the cluster and meta-cluster sizes. A medical doctor assessed the biological relevance of the resulting meta-clusters based on heat maps similar to the one shown in Figure 1A and we present the result for the clustering that was most biologically meaningful.

The final 12 meta-clusters contained a total of 18,374,011 cell events. Supplementary figure 3 shows numbers of events excluded from the analysis.

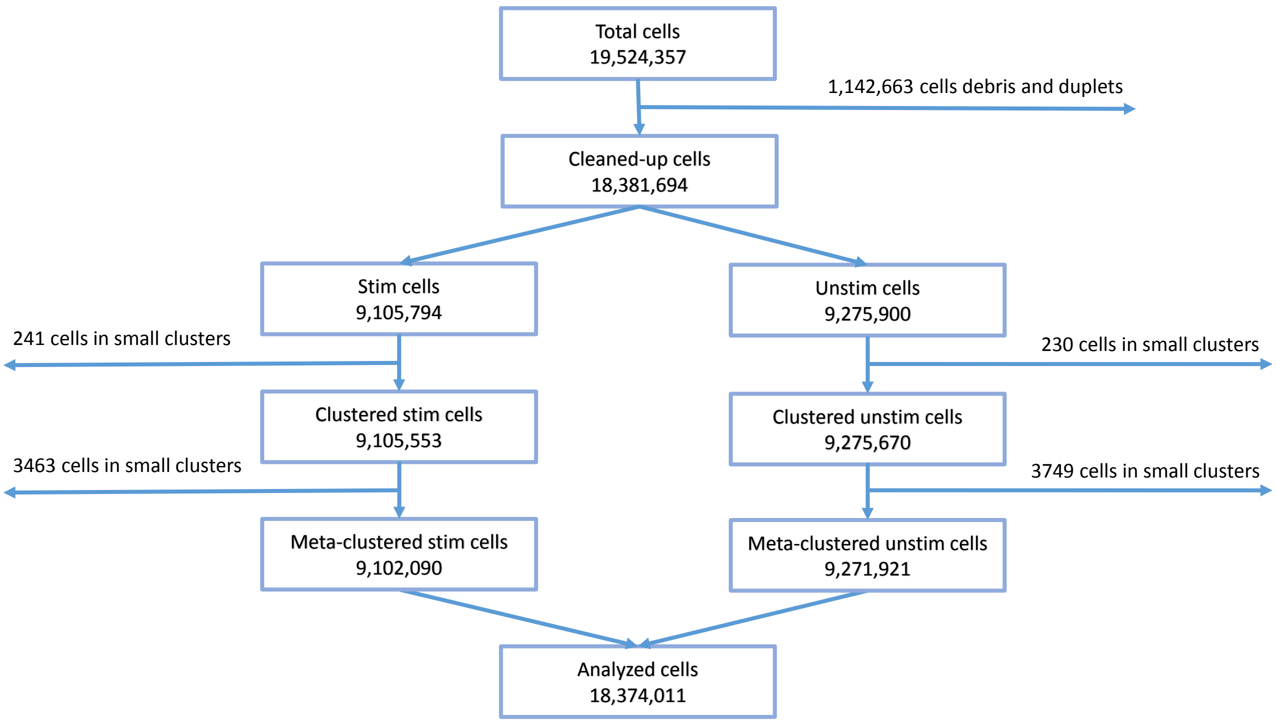

Supplementary figure 3: Cell inclusion/exclusion numbers in the NM2B algorithm.

The classification method we used for determining if individuals were patients or healthy donors was lasso regression. We used the following functional markers as features for classifying individuals as patients or controls: "142Nd_Caspase3", "156Gd_p-p38", "152Sm_p-Akt", "167Er_p-cJun", "166Er_p-NFkB", "164Dy_IkBa", "155Gd_CD120a", "165Ho_CD120b", "150Nd_CD86". For each combination of meta-cluster and functional markers, we calculated the median and 90% quantile in stimulated and basal cells. We also calculated the arcsinh ratios (difference between arcsinh-tranformed data) of functional markers between stimulated and basal cells. This gave us a total of 432 features (12 meta-clusters, 9 functional markers, 2 basal/ratio, 2 median/90% quantile). A principle component analysis of all 648 features colored by classification outcome (patient or healthy donor) is shown in Figure 1D. In addition to using all features (combined), we also tested a model based on the 216 features given by basal variables only (basal) and a model based on the 216 features given by the ratios only (ratio).

We fitted a lasso regression model to the data using double leave-one-out cross validation as follows. We used a first level of leave-one-out cross-validation to find the prediction accuracy. Inside the first level of cross-validation, we performed another level of leave-one-out cross-validation to find the L1-penalty coefficient λ for the lasso regression resulting in the minimum mean cross-validated error. We then used the L1-penalty coefficient λ found in the second level of cross-validation to predict if the individual left out in the first cross-validation level was a healthy donor or a rheumatic arthritis patient. To calculate the cross-validation accuracy we compared the prediction with the actual status of the individual that was left out. Repeating this for all individuals, we could calculate a mean cross-validation accuracy. Figure 1C shows a summary of cross-validation accuracies in the three different tested models, including the area under the ROC curve (AUC).

Pipeline 2: CITRUS algorithm

For this analysis, the cluster identification, characterization, and regression tool CITRUS in cytobank.org was used.

A. Import of normalized, concatenated and debarcoded .fcs to cytobank.org
B. Manual clean-up of raw data with the steps “time”, “singlets”, “tight”, “residual”, “center” and “live cells” as demonstrated in supplementary figure 4 removal of time intervals affected by injector clogging in the cytometer, followed by commonly applied biaxial clean-up of “singlets”, “tight” and “live cells”

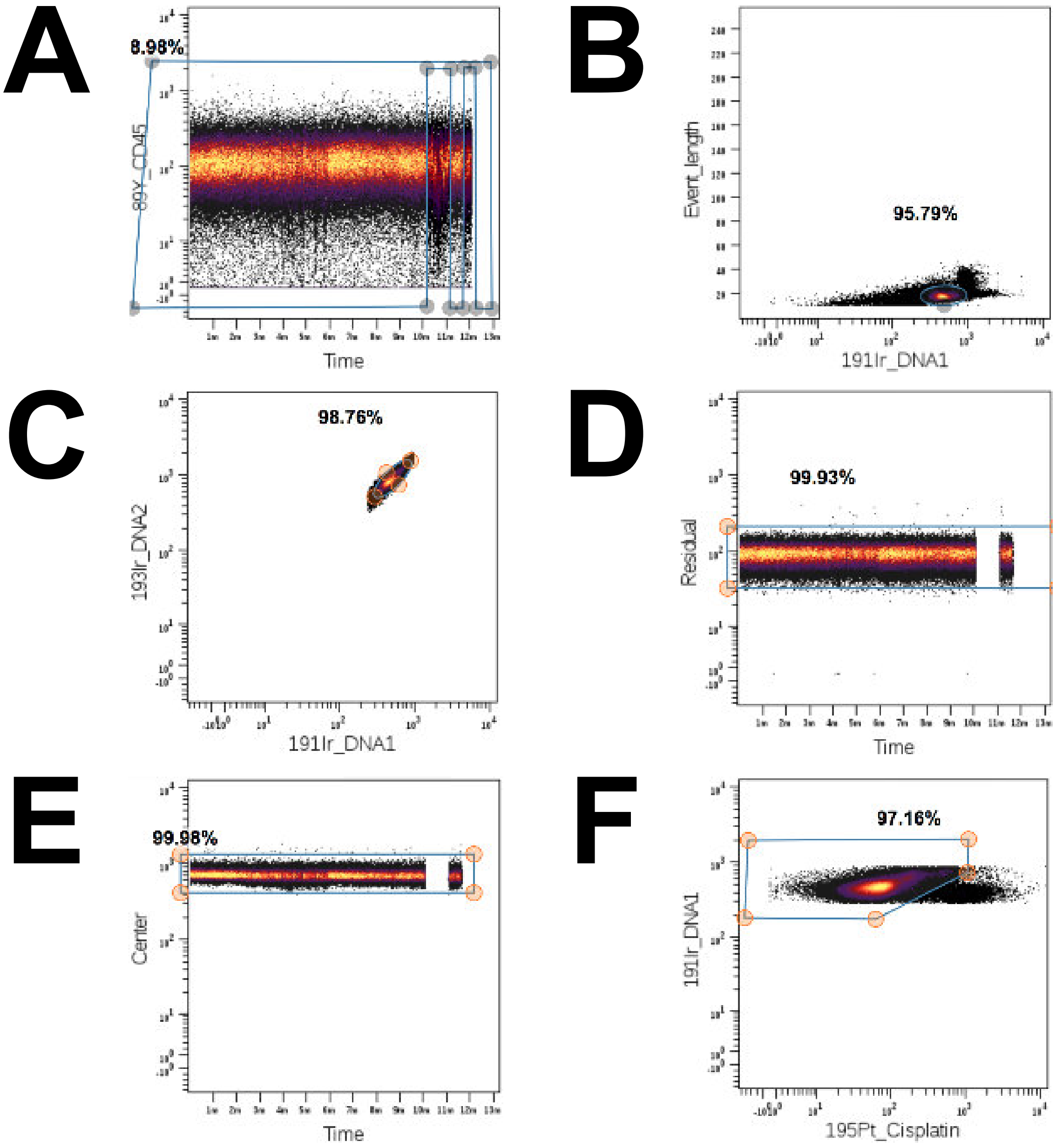

Supplementary figure 4: Manual clean-up by hierarchical biaxial biaxial gating.”Time” (removal of time intervals affected by injector clogging in the cytometer (A)), “Single cells” (B), “tight” (C), “residual” (D), “center” (E) and “live cells” (F)

C. Downsampling to 50k events per donor/patient and condition
D. CITRUS analysis with 3 repetitions on the same dataset (but with random downsampling from 50k to 10k events) with the following settings:

Compensation:	File-internal
Events per file:	10000
Minimum cluster size:	1
Cross validation folds:	5
False discovery rate:	5
Association models:	["pamr"]
Clustering characterization:	medians
Event sampling method:	equal
Normalize scales:	false
Plot theme:	white

Clustering channel short names: ["Bi209Di", "Er170Di", "Eu151Di", "Gd160Di", "Nd145Di", "Nd146Di", "Nd148Di", "Sm147Di", "Tb159Di", "Tm169Di", "Yb176Di", "Yb174Di"]
Median statistic channel short names: ["Dy164Di", "Er166Di", "Er167Di", "Gd155Di", "Gd156Di", "Ho165Di", "Nd142Di", "Nd150Di", "Sm152Di"]

Pipeline 3: Manual analysis

A. Import of normalized, concatenated and debarcoded .fcs to cytobank.org
B. Manually clean-up by commonly practiced biaxial gating of DNA1 versus event length (“single cells”), DNA1 versus DNA2 (“tight”) and Cisplatin versus DNA2 (“live cells”)

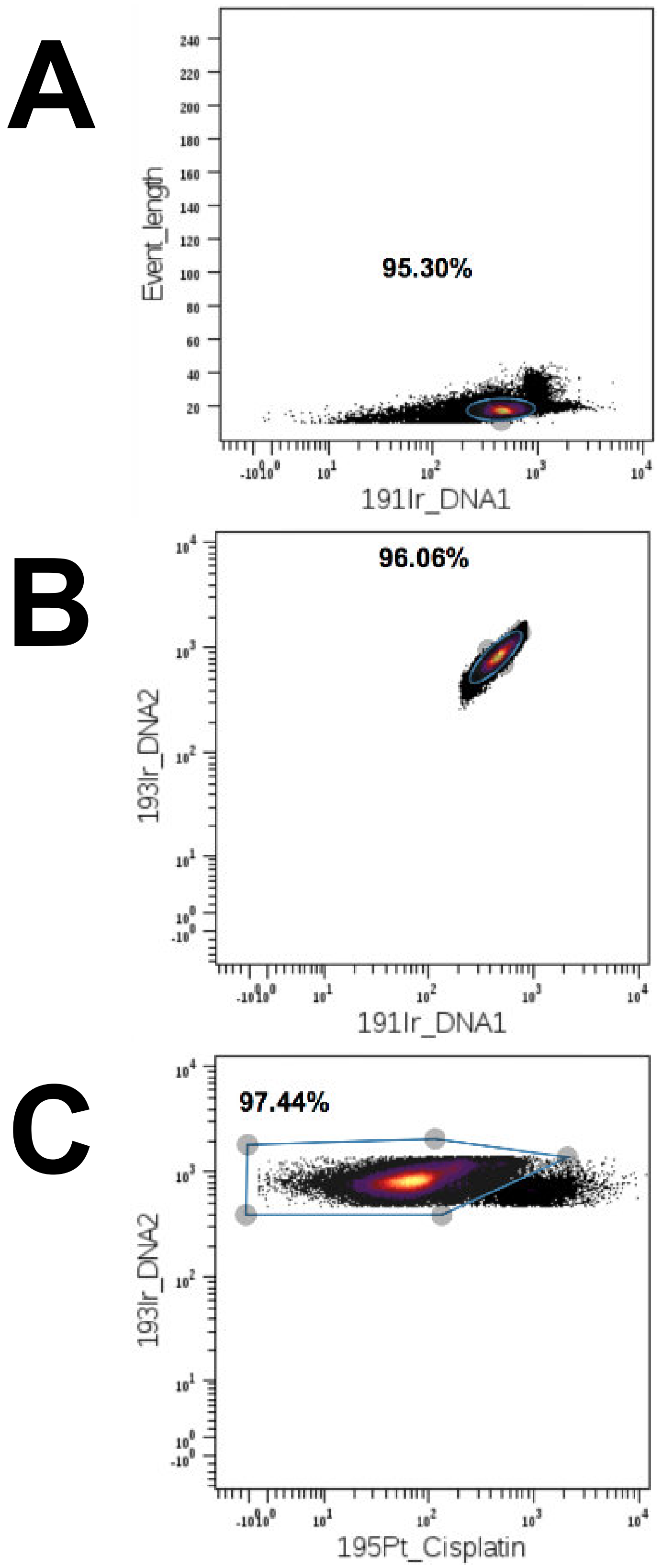

Supplementary figure 5: Manual clean-up by hierarchical biaxial gating.“Single cells” (A), “tight” (B), “live cells” (C).

C. Separate visualization of t-stochastic neighbor embedding (viSNE) plots were produced for each donor/patient, each plot consisting of 50k unstimulated and 50k TNF-stimulated live cell events. viSNE plots were based on phenotyping markers and run with 1000 iterations, perplexity of 50 and a theta of 0.5
D. Gating of cell subpopulations was performed manually on viSNE plots: B cells, CD4 naïve T cells, CD4 memory T cells, CD8 naïve T cells, CD8 memory T cells, CD3 CD4^{neg} CD8^{neg} T cells, NK T cells, NK cells, classical monocytes, myeloid dendritic cells, plasmacytoid dendritic cells

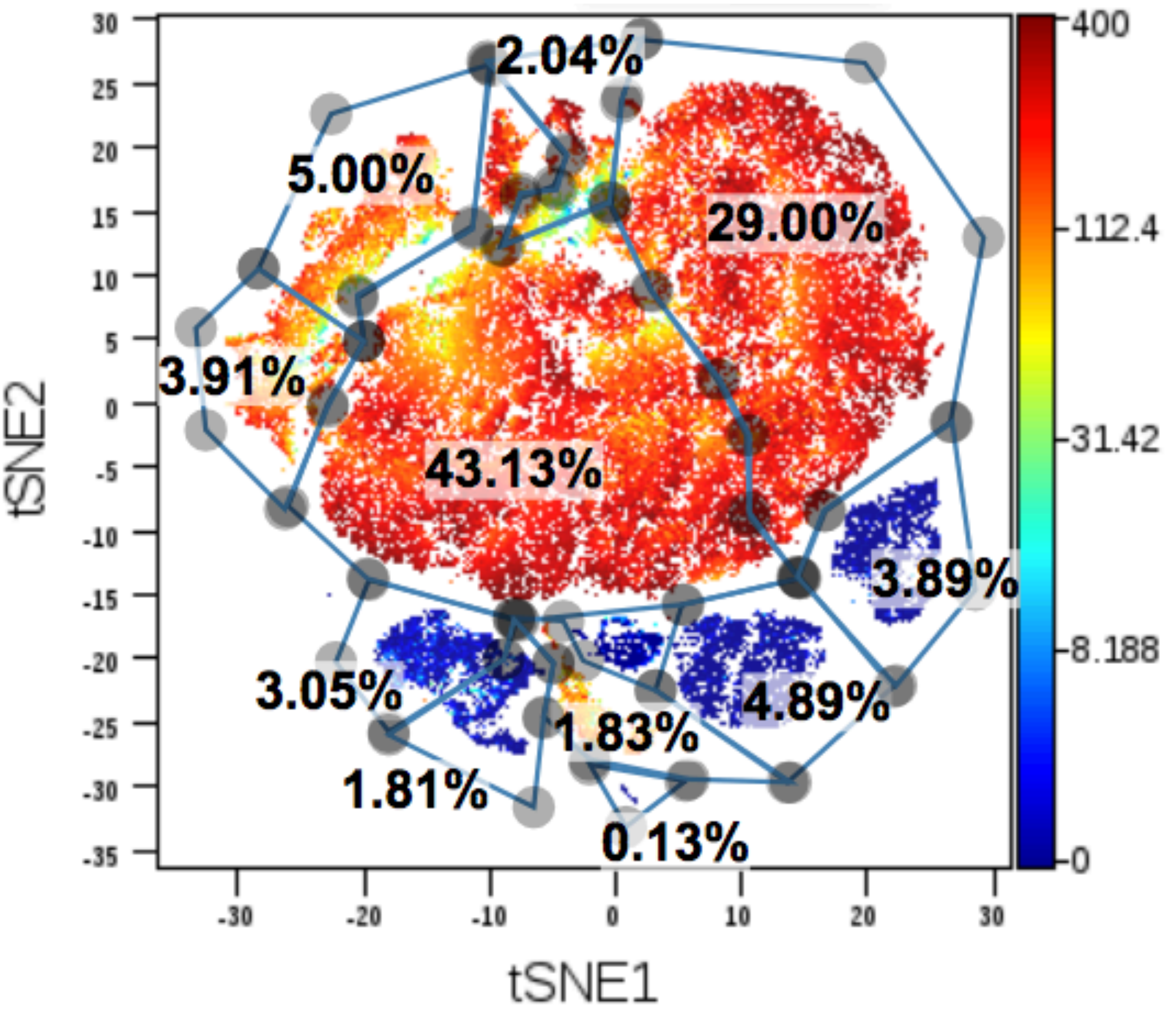

Supplementary figure 6: viSNE plot from one donor with gated cell subsets, color/heat indicates dual count expression of CD3 as indicated on the right-hand scale.

E. The same cell subpopulations from all donors and patients, both unstimulated and TNF-stimulated, were exported into a new cytobank experiment and compared side-by-side, especially concerning the expression of functional markers unstim-stim and healthy-sick
F. 75th percentiles and arcsinh ratios were exported to GraphPad Prism and analyzed applying non-parametric Mann-Whitney tests. Correction for multiple comparisons was not conducted due to the explorative character of this study

RESULTS

Pipeline 1: NM2B algorithm

Principle component analysis (PCA) was carried out as a part of this algorithm on all features (12 meta-clusters and 9 functional markers) and on features resulting from Lasso regression (11 distinct combinations of meta-clusters and functional markers).

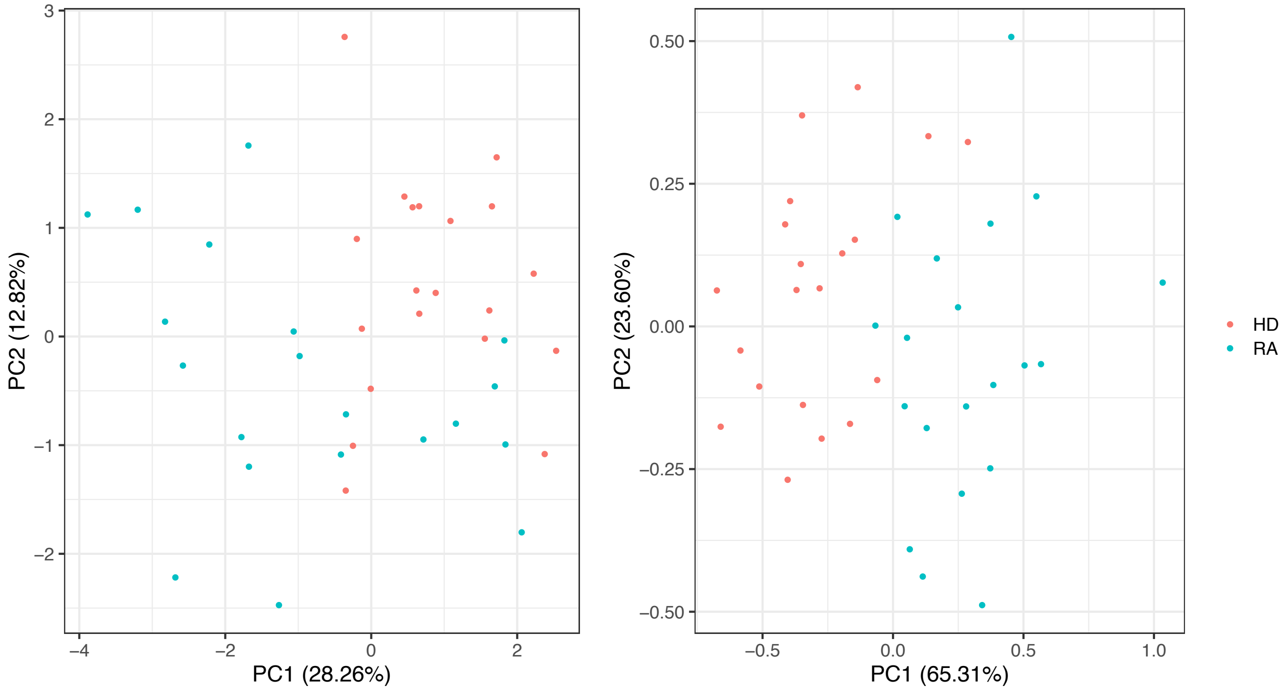

Supplementary figure 7: PCA of all features (left) and with features after Lasso regression (right), basal model.

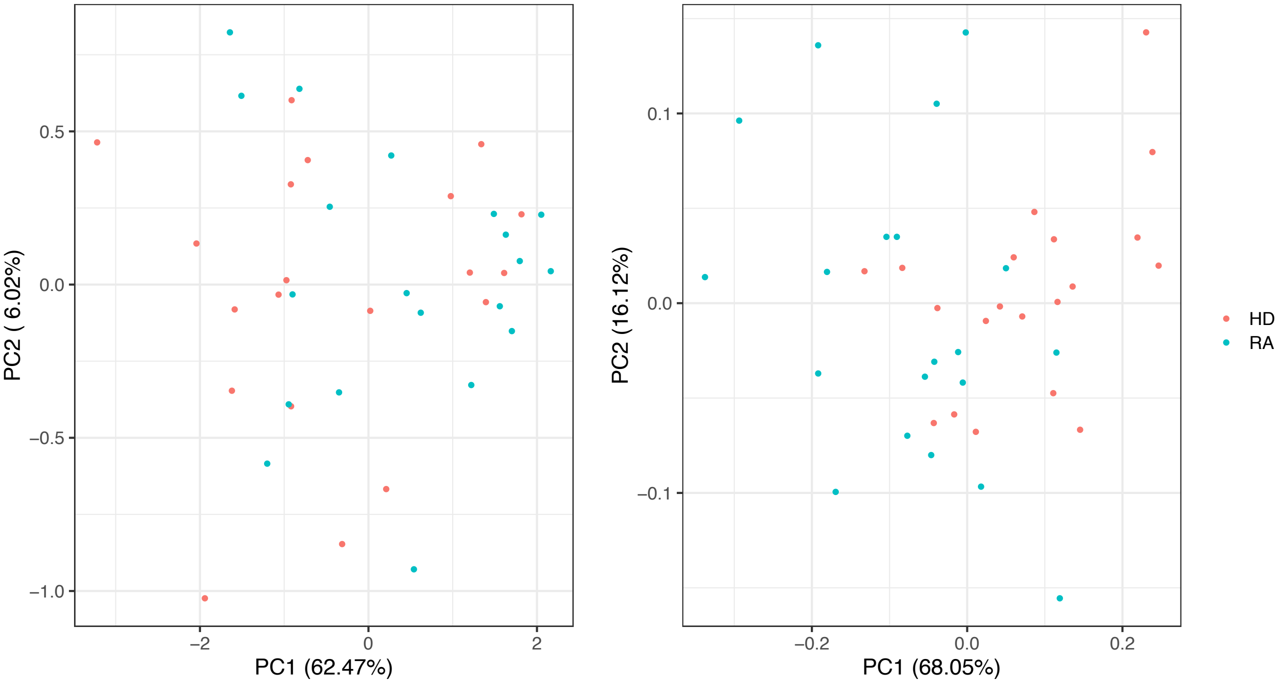

Supplementary figure 8: PCA of all features (left) and with features after Lasso regression (right), <u>ratio</u> model.

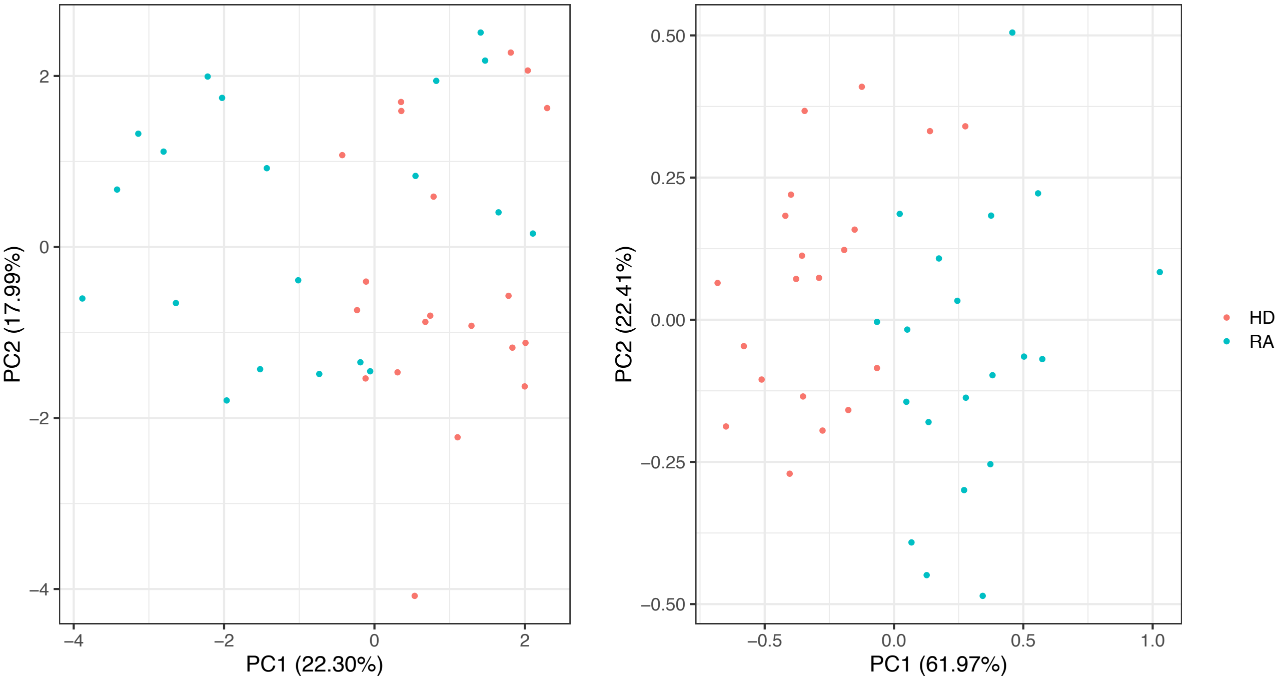

Supplementary figure 9: PCA of all features (left) and with features after Lasso regression (right), <u>combined</u> model.

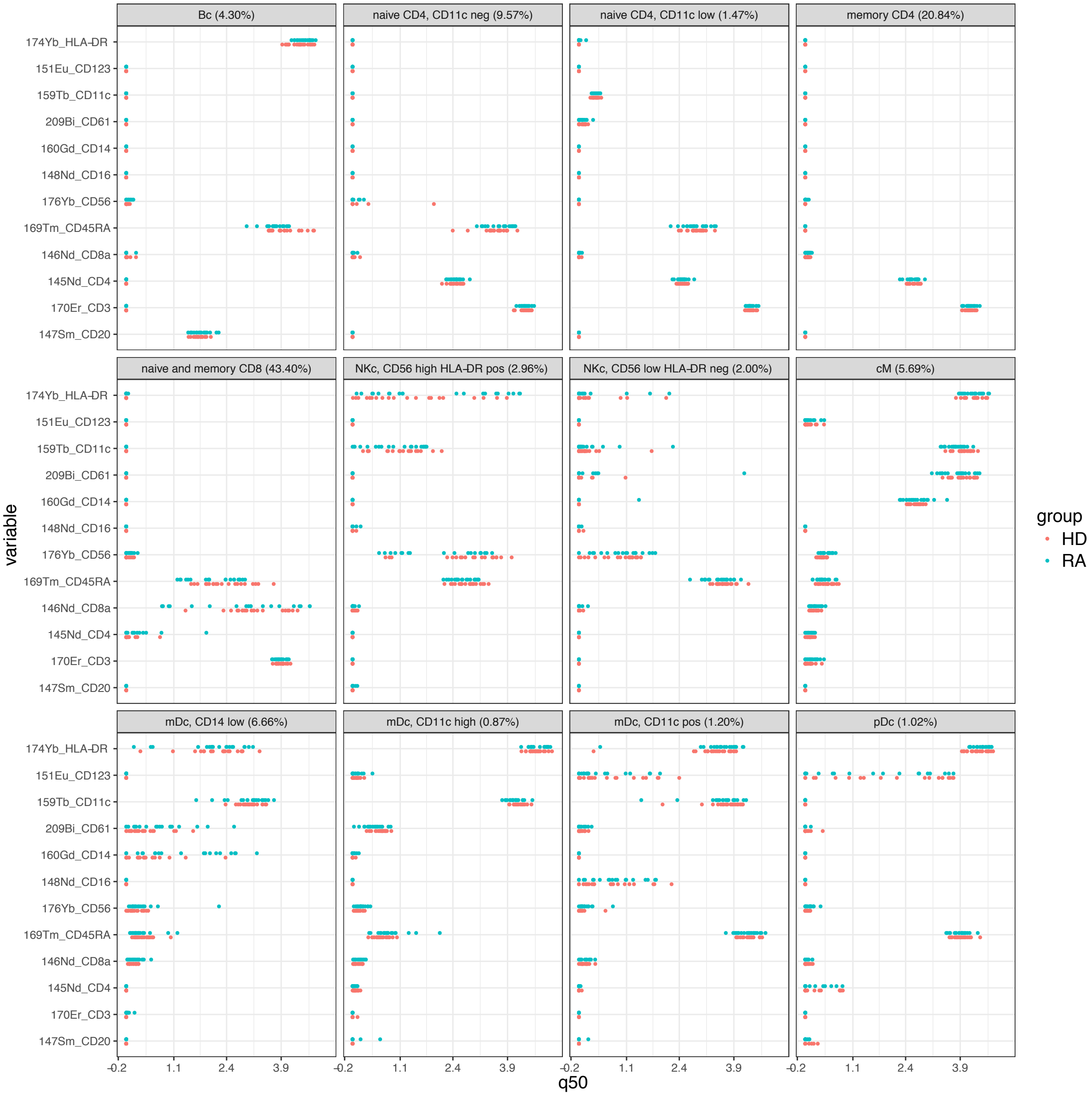

Supplementary figure 10: Differential expression of phenotyping markers in healthy donors (HD, red dots) versus rheumatoid arthritis patients (RA, turquoise dots) in 12 meta-clusters.

Pipeline 2: CITRUS algorithm

In the following, a summary of CITRUS settings and results from CITRUS analyses is presented. All four CITRUS analyses were carried out with the same settings.

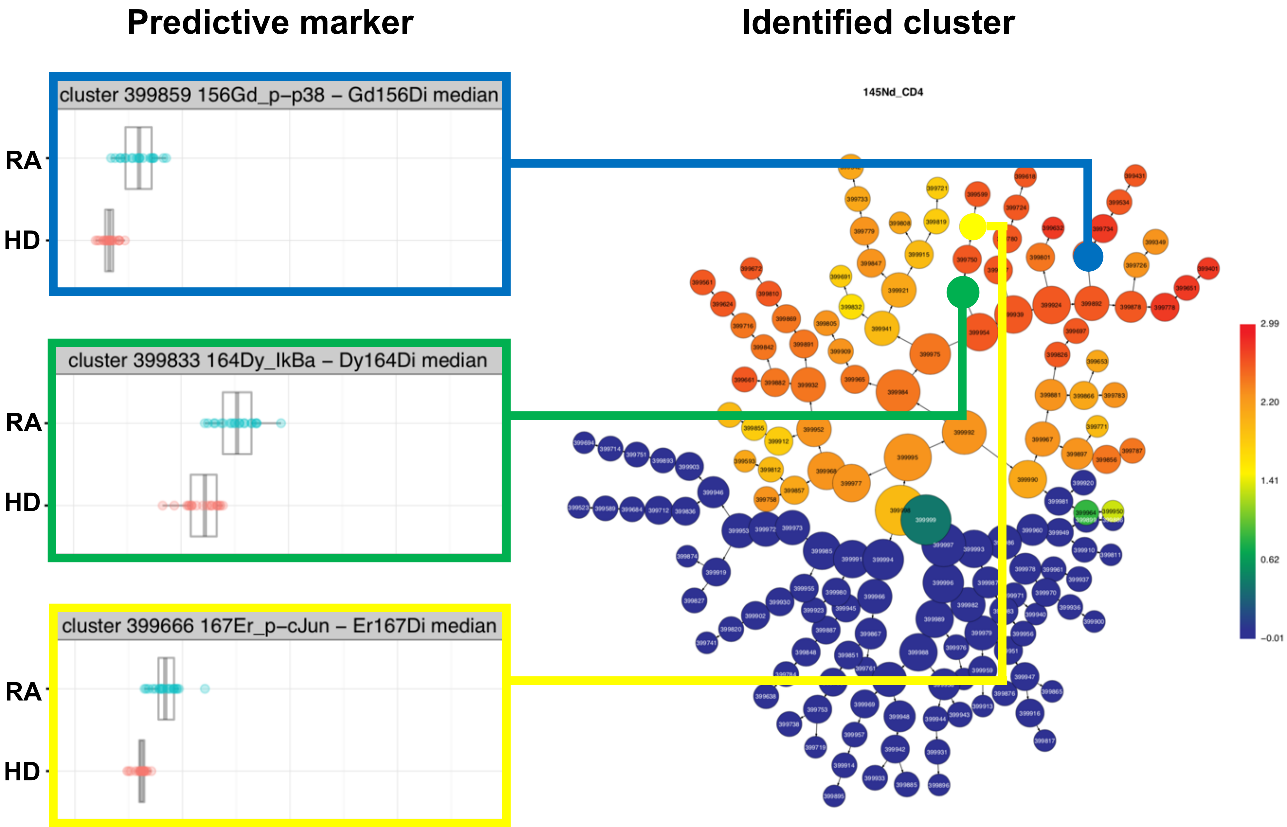

Supplementary figure 11: Exemplary CITRUS results. On the left side three functional markers (blue=p-p38, green=IkBa, yellow=p-cJun) identified as predictive markers by CITRUS, placed in the clusters they are belonging to on the right side: clustering by CITRUS, colored for CD4 expression.

	CITRUS analysis			
	**1**	**2**	**3**	**4**
**Predictive marker and cluster**	p-p38 in memory CD4 Tc<br>IkBa in memory CD4 Tc<br>p-cJun in memory CD4 Tc<br>p-NFkB in mDc<br>CD86 in cM	p-NFkB in mDc<br>p-p38 in memory CD 4 Tc<br>IkBa in memory CD4 Tc<br>p-cJun in memory CD4 Tc<br>CD86 in cM	p-p38 in memory CD4 Tc<br>p-NFkB in mDc<br>IkBa in memory CD4 Tc<br>p-cJun in mDc/cM<br>CD86 in cM/mDc	p-p38 in memory CD4 Tc<br>p-cJun in memory CD4 Tc<br>IkBa in memory CD4 Tc<br>p-NFkB in cM<br>n.n.

Supplementary table 10: Interpretation of results from 4 CITRUS analyses for the 5 functional markers of strongest predictive value. Modeling in analysis 4 resulted in only 4 predictive markers.

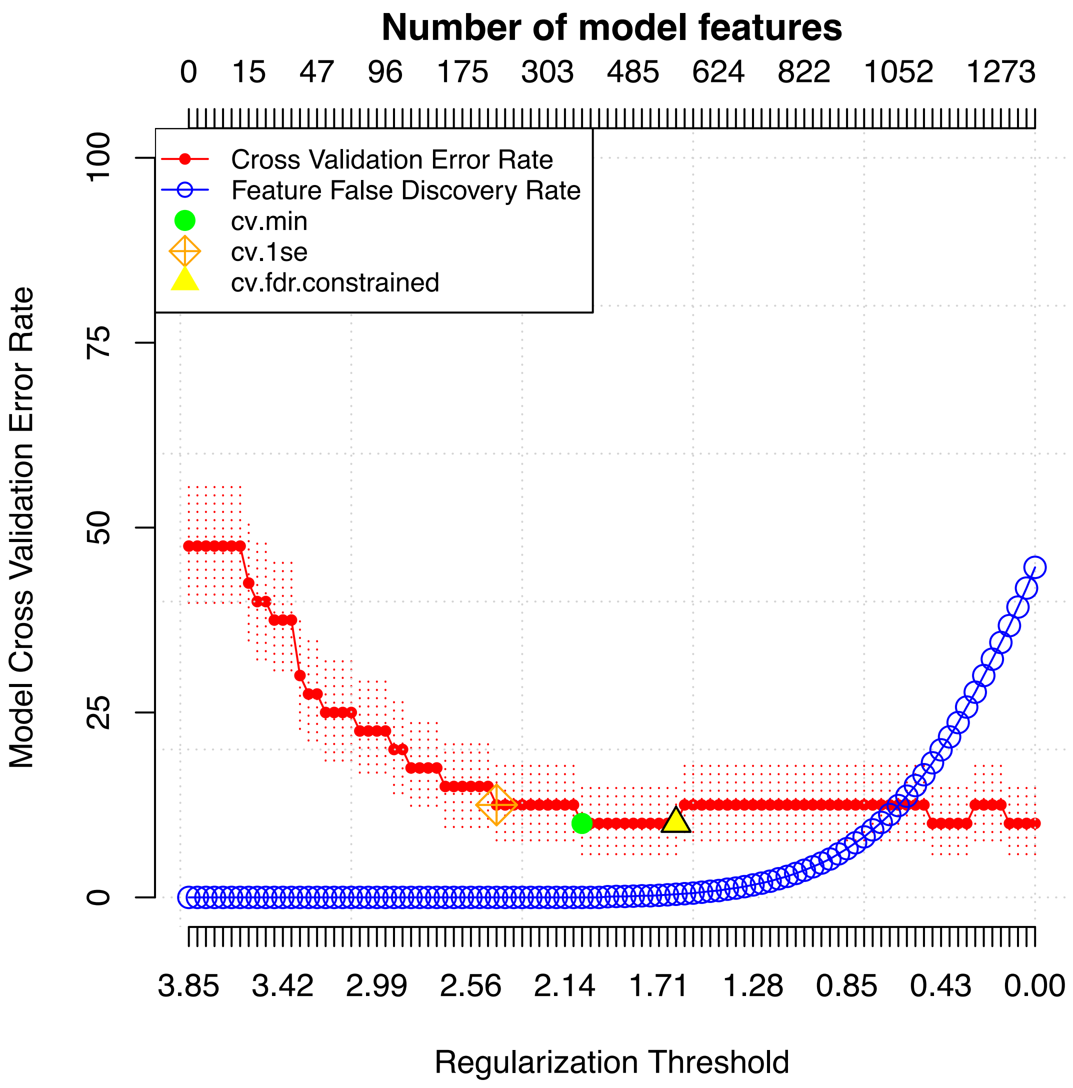

Supplementary figure 12: Model cross validation error rate from one out of four CITRUS runs.

Pipeline 3: Manual analysis

We compared both basal/unstimulated and TNF-stimulated expression and also arcsinh ratios (stim/unstim) of functional markers in cell subsets from RA patients and healthy donors. In general, differences between health and disease were weaker for functional markers after TNF-stimulation. Only data for unstimulated samples will be presented here.

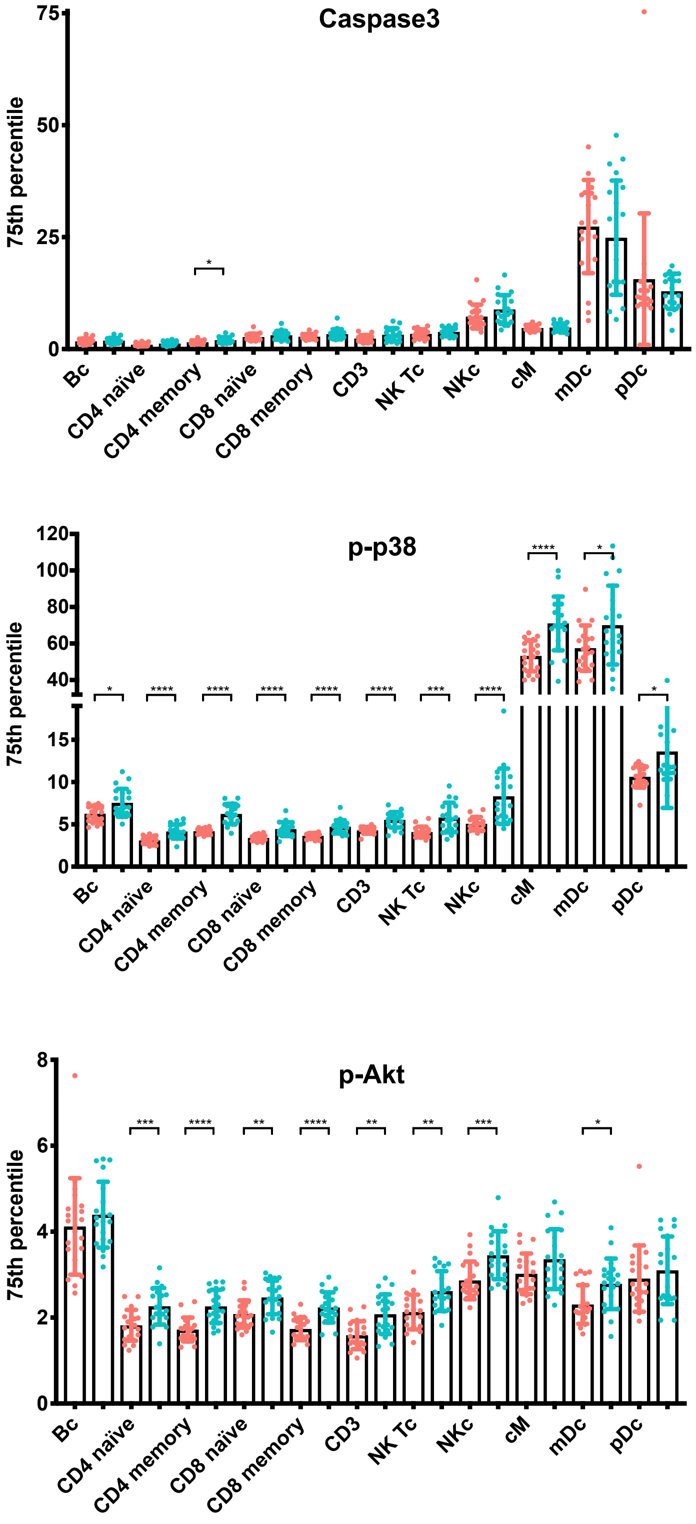

Supplementary figure 13: Differential expression of markers Caspase3, p-p38 and p-Akt in unstimulated cell subsets from healthy donors (pink) and RA patients (turquoise). Non-parametric Mann-Whitney testing with level of significance (* $p<0.05$, ** $p<0.01$, *** $p<0.001$ **** $p<0.0001$) without correction for multiple comparisons.

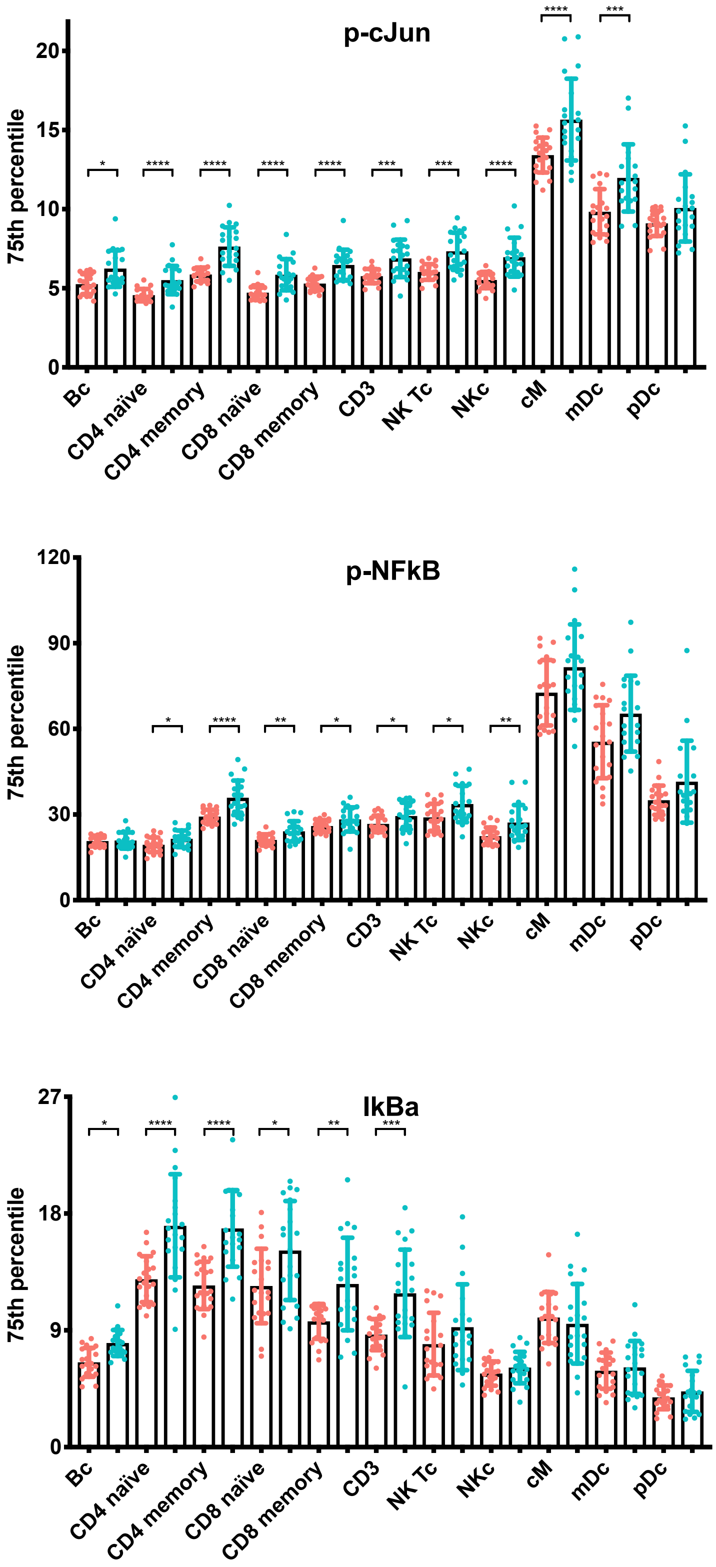

Supplementary figure 14: Differential expression of markers p-cJun, p-NFkB and IkBa in unstimulated cell subsets from healthy donors (pink) and RA patients (turquoise). Non-parametric Mann-Whitney testing with level of significance (* p<0.05, ** p<0.01, *** p<0.001 **** p<0.0001) without correction for multiple comparisons.

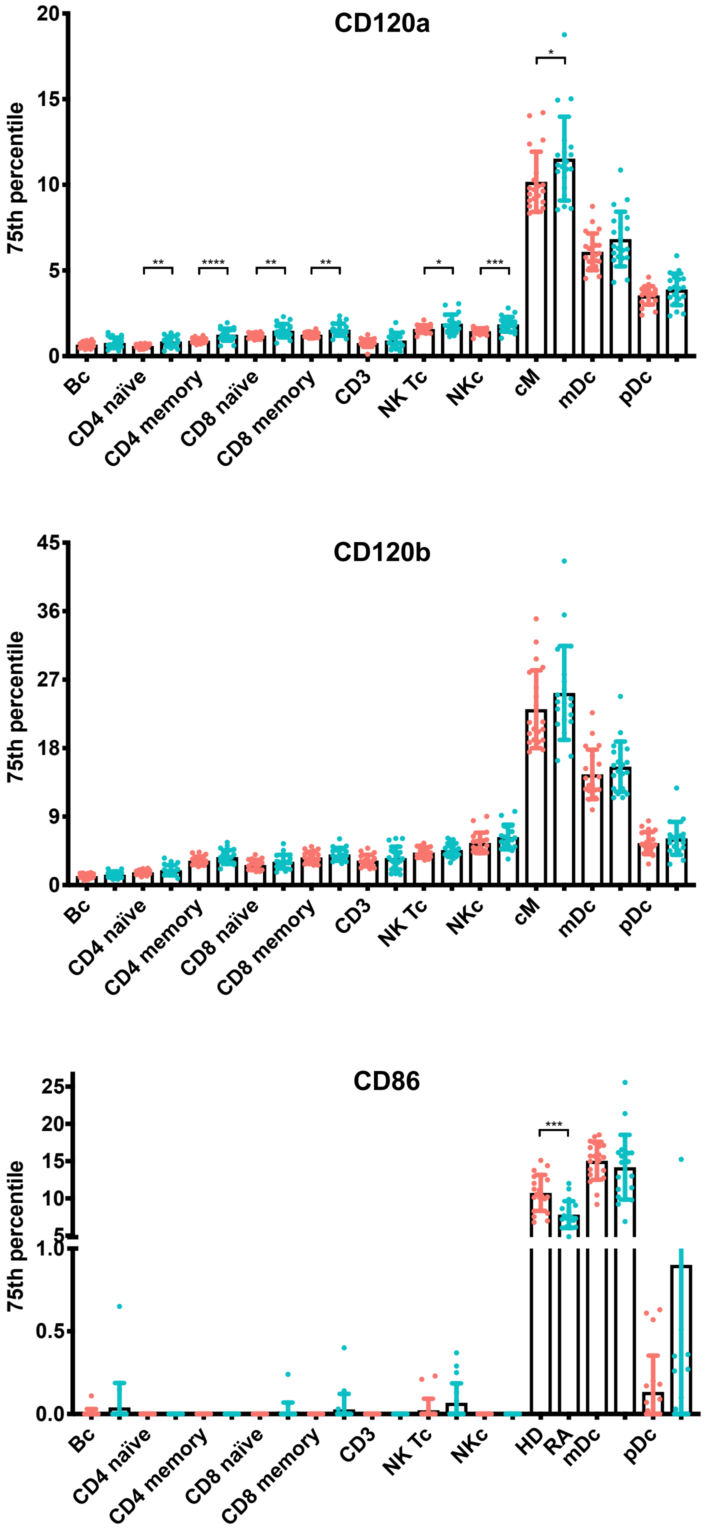

Supplementary figure 15: Differential expression of markers CD120a, CD120b and CD86 in unstimulated cell subsets from healthy donors (pink) and RA patients (turquoise). Non-parametric Mann-Whitney testing with level of significance (* p<0.05, ** p<0.01, *** p<0.001 **** p<0.0001) without correction for multiple comparisons.

frontiers
